# Evaluating myxovirus resistance protein A-based rapid testing combined with pathogen sequencing for arboviral and incidental viral infection surveillance in Senegal

**DOI:** 10.1128/spectrum.03392-25

**Published:** 2026-06-03

**Authors:** Mouhamed Kane, Serge Freddy Moukaha Doukanda, Safietou Sankhé, Bocar Sow, Marie Henriette Dior Ndione, Moundhir Mhamadi, Madeleine Dieng, Seynabou Mbaye Ba Souna Diop, Seynabou Seye, Maimouna Mbanne, Oumar Faye, Mamadou Aliou Barry, Pape Mbacke Sembene, Cheikh Loucoubar, Gamou Fall, Amadou Diallo, Cheikh Tidiane Diagne, Ndongo Dia, Moussa Moise Diagne

**Affiliations:** 1 Virology Department, Institut Pasteur de Dakar 89248, Dakar, Senegal; 2 Animal Biology Department, Université Cheikh Anta Diop de Dakar 89230 , Dakar, Senegal; 3 Public Health Department, Institut Pasteur de Dakar 89248, Dakar, Senegal; 4 Public Health and Preventive Medicine Department, Université Cheikh Anta Diop de Dakar 89230 , Dakar, Senegal; 5 DIATROPIX Unit, Institut Pasteur de Dakar 89248, Dakar, Senegal; 6 Epidemiology, Clinical Research and Data Science Department, Institut Pasteur de Dakar 89248, Dakar, Senegal; Cleveland Clinic, Cleveland, Ohio, USA; Debre Markos University, Debre Markos, Ethiopia

**Keywords:** arboviruses, rapid diagnostic test, host-response biomarkers, myxovirus resistance protein A, viral sequencing, pathogen surveillance, dengue, chikungunya, interferon-stimulated genes

## Abstract

Accurate differentiation between viral and bacterial infections remains challenging in resource-limited, arbovirus-endemic settings, leading to antibiotic misuse and diagnostic uncertainty. Myxovirus resistance protein A (MxA), an interferon-induced host biomarker, may offer a pathogen-agnostic approach to improve rapid diagnosis and clinical triage. We evaluated the performance of an MxA rapid diagnostic test (RDT) using archived samples from febrile patients collected during dengue virus (DENV) and chikungunya virus (CHIKV) outbreaks in Senegal. We tested 171 blood samples from patients with acute febrile illness using an MxA RDT and RT-qPCR for DENV and CHIKV. Samples with discordant results (MxA-positive and RT-qPCR–negative) underwent metagenomic and hybrid-capture Illumina-based sequencing to detect missed infections. Sequencing data were analyzed using maximum-likelihood phylogenetics to assess viral lineage placement. The MxA RDT demonstrated moderate-to-high sensitivity (70.0%–85.1%, depending on virus) and moderate specificity (70.2%) for detecting primary arboviral infections. Among discordant samples, sequencing revealed previously missed pathogens, including DENV serotype 3 (genotype III), Parvovirus B19 (B19V), and Torque teno virus (TTV). Detection of B19V and TTV highlights the broader clinical utility of host-response biomarkers to uncover unexpected viral pathogens in high-diversity settings. MxA’s longer persistence than viral RNA enables detection of recent infections missed by PCR. Combined with sequencing, this broadens the diagnostic window, improves clinical triage, and supports identification of underdiagnosed viruses. Future research should integrate MxA testing into routine clinical care and surveillance protocols to enhance outbreak responses in resource-limited regions.

**IMPORTANCE:**

Timely and equitable viral diagnosis is vital in outbreak-prone regions where advanced laboratories are scarce. This study shows how a simple, rapid test for the host biomarker myxovirus resistance protein A can provide real-time detection of viral infections such as dengue and chikungunya, even in remote or frontline health centers. When paired with pathogen sequencing, the test also uncovers infections that standard PCR may miss. This integrated approach demonstrates how field-deployable diagnostics can operate both during and between epidemics, strengthening outbreak preparedness, improving patient triage, and advancing laboratory equity worldwide.

## INTRODUCTION

Acute febrile illness remains a diagnostic blind spot, especially in low-resource and outbreak settings. Viral and bacterial infections often present with overlapping symptoms, and empiric antibacterial therapy is frequently prescribed “just in case,” fueling antimicrobial resistance  (1). Biomarkers in current use, C-reactive protein (CRP) and procalcitonin (PCT), rise predominantly in bacterial sepsis but lack specificity, so the absence of elevation may suggest, yet cannot confirm, a viral etiology (2, 3). A host-derived marker that rises positively in response to viral infection could transform triage decisions and antibiotic stewardship.

Myxovirus resistance protein A (MxA) is a type I interferon (IFN)-stimulated, dynamin-like GTPase with broad antiviral activity and negligible induction by bacteria. MxA is upregulated within 1–2 h of interferon signaling, peaks by 16–24 h, and can remain elevated for more than 7 days in acute arboviral infections, potentially outperforming direct viral detection in patients with delayed presentation (4, 5).

Clinical studies in children and adults showed diagnostic efficacy of MxA in distinguishing between viral and bacterial infections (6, 7). Point-of-care formats that read MxA alone or in combination with CRP (e.g., FebriDx) deliver results in <15 min and have demonstrated utility during COVID-19 surges and seasonal respiratory outbreaks (8).

Despite this evidence, MxA has rarely been evaluated in arboviral epidemics. Dengue virus (DENV) and chikungunya virus (CHIKV) account for millions of cases annually in the tropics, including recent outbreaks in Senegal (9, 10). While CHIKV generally provokes a robust interferon response (11), DENV encodes IFN antagonist proteins (12) and may blunt MxA induction, raising concerns about test sensitivity in dengue. No field evaluation has yet benchmarked an MxA rapid diagnostic test (RDT) against well-characterized DENV and CHIKV clinical samples, nor explored how MxA concentrations correlate with viral load and days since symptom onset in these settings.

The expression of MxA protein has also been observed in HIV infections (13), as well as in RSV and rotavirus infections (6). The human MxA protein is known to inhibit various Bunyaviruses: LACV (Orthobunyavirus); CCHFV and Dugbe virus (Nairovirus); RVFV (Phlebovirus); and HTNV, PUUV, and TULV (Hantavirus) (14–18).

MxA-sensitive viruses include members of the bunyaviruses, orthomyxoviruses, paramyxoviruses, rhabdoviruses, togaviruses, picornaviruses, and hepatitis B virus (19–22).

MxA is induced as early as 1.2 h post-infection. The protein reaches peak concentrations at 16 h and remains elevated with increased IFN, with a half-life of around 2.3 days (23). Human MxA protein has been used as a biomarker to distinguish viral from bacterial infections in studies by Kawamura et al. Using sandwich-type enzyme-linked immunosorbent assay (ELISA) with monoclonal antibodies targeting MxA’s GTP-binding domain, the study showed that MxA offered superior sensitivity and specificity compared to other markers such as peripheral white blood cell count and serum CRP concentration, highlighting its value as an indicator of viral infection (24).

RDTs are vital for early case detection, surveillance, and guiding treatment. However, their development and implementation are hindered by insufficient funding and low investment from industry, especially in low-income countries, despite WHO recommendations (25). In 2023, Africa faced 180 public health emergencies, with 90% linked to infectious diseases and 75% to zoonotic origins. Effective testing capacity is crucial for the timely and accurate identification of these pathogens. Strengthening diagnostics is key to preventing the spread of epidemic-prone diseases (26).

This study was conducted to evaluate and validate the performance of the MxA-based rapid diagnostic test and ELISA using human whole blood samples, with the objective of reinforcing viral diagnostic capabilities and surveillance systems in the context of public health emergency preparedness.

## MATERIALS AND METHODS

### Study design and sample panels

This evaluation was conducted retrospectively on archived clinical blood samples from suspected arboviral cases during two epidemic surveillance efforts: a chikungunya outbreak in Kédougou (10) and a dengue outbreak in Dakar (2024). Initial diagnostic classification was based on RT-qPCR, and the study panel included (i) DENV-positive cases, (ii) CHIKV-positive cases, and (iii) double-negative cases (negative for both DENV and CHIKV by RT-qPCR) (27, 28). Available clinical metadata included age, sex, and reported date of symptom onset when documented in routine investigation forms; metadata completeness varied across specimens due to outbreak-response conditions.

Following initial diagnostic testing, samples were transferred to the WHO Collaborating Center for Arboviruses and Hemorrhagic Fever Viruses (CRORA) at the Institut Pasteur de Dakar for confirmatory laboratory analyses, including PCR and ELISA. All samples were stored at –80°C and later used for MxA-based rapid diagnostic testing and ELISA experiments. For this study, samples were thawed once under a standardized workflow, gently mixed, and aliquoted to minimize freeze–thaw variability before MxA RDT and ELISA testing. In this malaria-endemic setting, pan-Plasmodium conventional PCR was performed on a subset of specimens with sufficient residual volume to assess potential parasitic confounding.

Panels were pre-defined according to RT-qPCR status, precluding full blinding at the panel-selection stage. Specimens were relabeled with study IDs, and MxA operators were blinded to individual RT-qPCR results. Testing followed manufacturer workflows, with duplicate measurements and instrument-based RDT readout.

### MxA rapid diagnostic test

The MxA RDT was performed using the Bi-VirTest kit (BioVendor–Laboratorní medicína, Brno, Czech Republic, reference: BI005-10), according to the manufacturer’s instructions. The assay uses specific monoclonal antibodies to detect the MxA protein, leveraging gold nanoparticle-conjugated lateral flow technology. All consumables and reagents necessary for the assay were provided with the kit. For each test, six drops of lysis buffer were first added to the container of the empty uniSampler. Using the capillary-equipped cap, the whole blood sample was aspirated and inserted into the container. The uniSampler device was then shaken to lyse the sample. Subsequently, two drops of the lysed sample were applied to the sample well of the RDT cassette. After an incubation of 4–5 min, two drops of wash buffer were added to the same well. The result was read after 11 min using the Bi-Reader. Each sample was tested in duplicate. RDT results were recorded both qualitatively (positive/negative) and quantitatively based on the mean concentration (ng/mL) obtained across duplicates. Positivity was determined according to the manufacturer’s cut-off values (12 ng/mL). Duplicate measurements and instrument-based reading were used to reduce operator-dependent variability.

### MxA enzyme-linked immunosorbent assay

The MxA ELISA was conducted using the BioVendor Human MxA Protein ELISA kit (BioVendor–Laboratorní medicína, Brno, Czech Republic, reference: RD194349200R) following the manufacturer’s instructions. Whole blood samples, previously used for the RDT assay, were diluted 1:10 with dilution buffer. Six standards (12–0.375 ng/mL) were prepared by serial dilution. A total of 100 µL of standards and diluted samples were added to the coated plate and incubated for 1 h at 25°C with shaking (300 rpm). After washing, 100 µL of Biotin-Labeled Anti-MxA Antibody was added, followed by Streptavidin-HRP (BD Biosciences Cat# 550946, RRID: AB_2868972) after a second incubation. Detection was performed using Substrate Solution, and the reaction was stopped after 20 min. Absorbance was read at 630 nm. All tests were performed in duplicate, and mean concentrations (ng/mL) were calculated. Positivity was defined using a cutoff of 10 ng/mL (Biovendor IFU). To support consistency across plates, each run included the full standard curve and duplicate measurements for all specimens, and concentrations were reported as the mean of duplicates.

### Sequencing and pathogen discovery

The corresponding sera from individuals who tested positive by MxA RDT but negative by RT-qPCR for DENV and CHIKV were selected for sequencing. Sequencing was performed after completion of MxA testing and, therefore, did not influence MxA readouts. Two complementary approaches were employed to maximize viral detection.

For the first approach, we employed a metagenomic sequencing protocol as previously described (29). In short, RNA was extracted and subjected to host ribosomal RNA (rRNA) depletion using the NEBNext rRNA Depletion Kit v2 (New England Biolabs; Cat# E7400). cDNA synthesis was performed using the SuperScript IV Reverse Transcriptase (Invitrogen, Thermo Fisher, Cat# 18090010; RRID: AB_2620444), and sequencing libraries were prepared using the Nextera XT DNA Library Preparation Kit (Illumina; Cat# FC-131-1096). Paired-end sequencing (2 × 150 bp) was carried out on an Illumina MiSeq platform using standard run procedures.

The second approach utilized a targeted hybrid capture workflow based on a standard enrichment protocol (10). Briefly, RNA was reverse-transcribed using random hexamers and SuperScript IV Reverse Transcriptase, followed by second-strand synthesis with Klenow Fragment DNA Polymerase (New England Biolabs; Cat# M0210). Double-stranded cDNA was purified with AMPure XP Beads (Beckman Coulter; Cat# A63881) and processed using the Twist Total Nucleic Acids Library Preparation Kit (Twist Bioscience; Cat# 101057), which includes enzymatic fragmentation, end-repair, adapter ligation, and indexing. Libraries were pooled and enriched using the Twist Comprehensive Viral Research Panel (Twist Bioscience; Cat# 105319), which contains approximately 1 million probes targeting over 3,000 viral genomes (30, 31), followed by a 16 h hybridization step. Sequencing was performed on an Illumina iSeq 100 platform using 150 bp paired-end reads.

Raw sequencing data (FASTQ files) from both workflows were analyzed using the CZ ID metagenomic platform (https://czid.org/). Sequences with >80% genome breadth and >10 × mean coverage were retained for downstream analysis. Viral contigs were identified using BLASTn, aligned with MAFFT v7.520 (32), and manually curated with AliView v1.28 (33). Phylogenetic trees were constructed with IQ-TREE v2.2.6 using ModelFinder for model selection and 1,000 bootstrap replicates and visualized with R 4.3.2 using ggtree 3.10.1 together with treeio 1.26.0, tidytree 0.4.6, and ggplot2 3.4.4 (34).

An overview of the diagnostic and sequencing workflow is provided in Fig. S1.

### Statistical analysis

Qualitative agreement between the MxA RDT and the ELISA was assessed by generating contingency tables comparing their qualitative results (positive/negative classifications). All diagnostic comparisons used the same pool of 84 specimens that were PCR negative for both DENV and CHIKV.

Positive predictive value (PPV) and negative predictive value (NPV) were calculated directly from the confusion matrices, and their 95% confidence intervals (*CIs*) were estimated with the Wilson score method (*n* = TP + FP for PPV, *n* = TN + FN for NPV) (35).

Agreement between MxA-RDT and MxA-ELISA cutoff-based positive/negative classifications was quantified using Cohen’s kappa, unweighted, as previously described (36).

Quantitative correlation between RDT MxA and ELISA MxA mean concentrations (ng/mL) was evaluated using both Spearman’s rank correlation coefficient and Pearson’s correlation coefficient. Spearman’s correlation assessed the monotonic relationship between the two assays, while Pearson’s correlation evaluated the linear relationship (37).

Comparisons of RDT MxA concentrations across RT-PCR-confirmed groups (CHIKV-positive, DENV-positive, and double-negative) were performed using the Kruskal-Wallis *H*-test for overall group comparison and pairwise Mann-Whitney *U* tests with Bonferroni correction (significance threshold *P* < 0.0167) for between-group comparisons (38). For samples stratified by viral load categories (high, intermediate, and low), similar Kruskal-Wallis tests were used to determine whether MxA levels varied significantly with viral burden.

Diagnostic performance of MxA concentrations was further evaluated through receiver operating characteristic (ROC) curve analysis (39). The area under the ROC curve (AUC) was computed to quantify the discriminatory ability of MxA concentrations to distinguish DENV- or CHIKV-positive infections from negatives. The optimal cut-off thresholds were determined using Youden’s *J* statistic (*J* = sensitivity + specificity − 1) (40). Cutoffs were derived on the study cohort and therefore require external validation in an independent data set. *CIs* for the AUC were estimated through bootstrap resampling (2,000 iterations) when appropriate. Analyses were conducted for (i) all viral infections (CHIKV or DENV) vs negative, (ii) CHIKV vs negative, and (iii) DENV vs negative samples.

The 95% *CIs* for sensitivity and specificity were calculated with the Wilson score method (*n* = TP + FN and *n* = TN + FP, respectively) (41). The 95% CI for the AUC was obtained by nonparametric bootstrapping with 2,000 random resamples of the cohort.

All statistical analyses were performed using Python (version 3.11.6), primarily leveraging the scikit-learn v1.4.1, and statsmodels v0.14.0 (42).

## RESULTS

### Study population and sample collection

The study included 171 archived specimens collected during a dengue outbreak in Dakar 2024 and a chikungunya outbreak in Kedougo (10); 67 samples were DENV-positive, 20 CHIKV-positive, and 84 were outbreak-period arbovirus-negative controls sampled concurrently. Power calculations assuming an expected MxA rapid test sensitivity (*P* = 0.80) and a 95% CI halfwidth (d ≤ 0.10) yielded a minimum of 62 virus-positive samples via *n* = \frac{z_{0.975}^{2} p(1−p)}{d^{2}}; the 87 positives exceeded this requirement.

### Qualitative and quantitative correlations between RDT and ELISA MxA

Of the 171 samples tested, two lacked ELISA results and were excluded from agreement analyses. Applying manufacturer’s cut-offs (RDT: 12 ng/mL; ELISA: 10 ng/mL), which confer ≥92% sensitivity and ≥95% specificity for the RDT and a 0.001 ng/mL limit of detection for the ELISA, we observed 83.4% qualitative agreement across 169 samples with paired results, with 141 concordant positive or negative results. The remaining 28 samples showed discordant results, typically around the assay-specific cut-offs, suggesting borderline expression levels or inter-assay variation. Cohen’s kappa was 0.67, indicating moderate-to-substantial agreement beyond chance.

**Fig 1:**
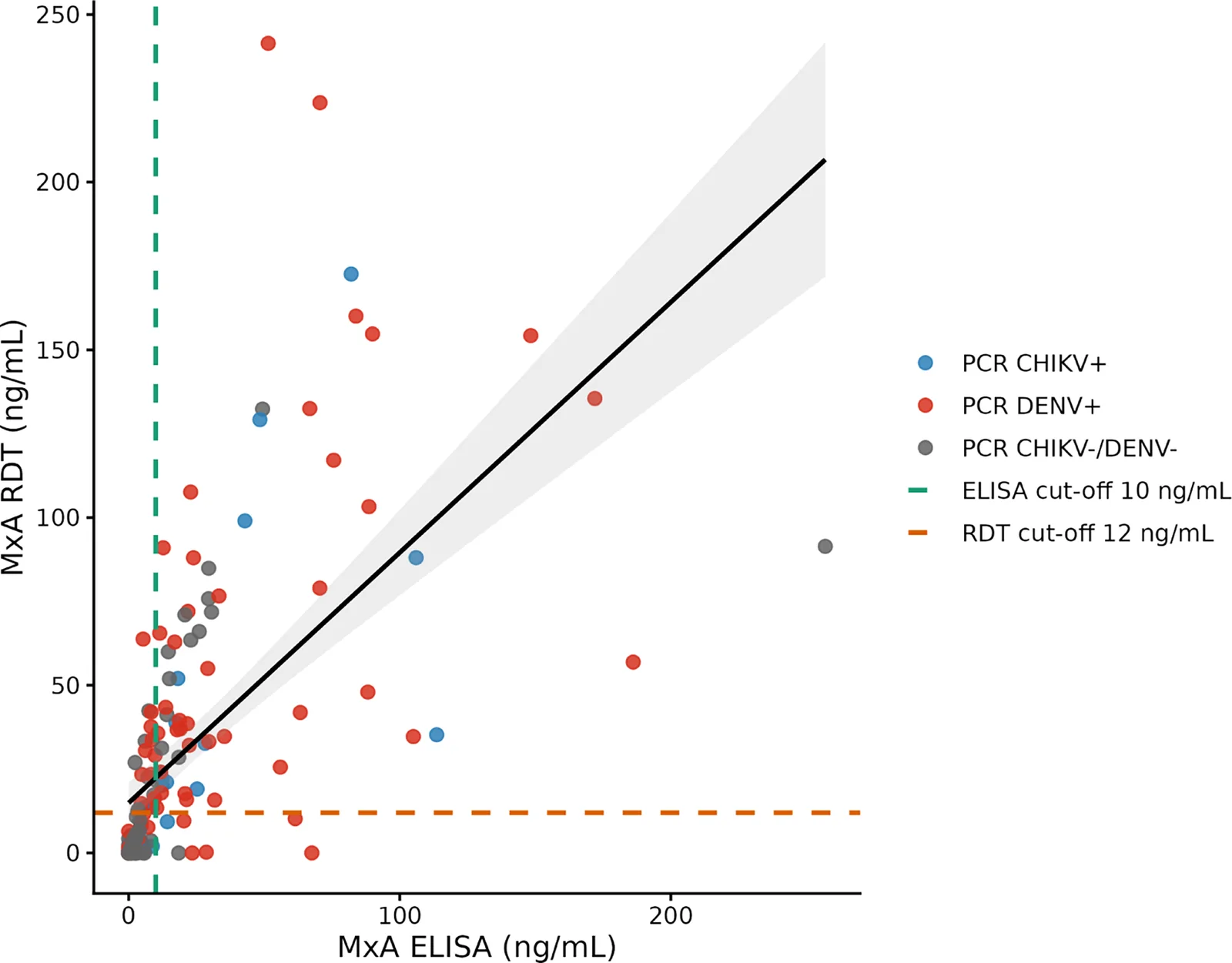
Correlation between MxA concentrations measured by RDT and ELISA. Each point represents a clinical sample with paired quantitative measurements (*n* = 169). There was a strong positive correlation between the two assays (Spearman *r* = 0.81, *P* < 0.0001). Dashed lines indicate positivity cutoffs (ELISA 10 ng/mL, RDT 12 ng/mL). Using these predefined cutoffs, qualitative agreement was 83.4%, and Cohen’s kappa was 0.670, indicating moderate-to-substantial agreement beyond chance.

Quantitatively, there was a strong positive correlation between RDT and ELISA measurements of MxA (Spearman *r* = 0.81, *P* < 0.0001). This correlation persisted across all diagnostic categories, indicating that despite differing formats and performances, both platforms reliably capture the interferon-stimulated MxA host response (Fig. 1; Table S1). PCR-confirmed DENV and CHIKV cases were clustered predominantly in the upper-right quadrant, exceeding both thresholds, while PCR-negative cases largely concentrated in the lower-left quadrant. The scatter around the thresholds also highlighted the value of using dual platforms or quantitative interpretation in ambiguous cases. These findings support the biological equivalence of the two detection formats, with the RDT potentially providing a suitable and scalable proxy in field-based applications where ELISA may not be feasible.

We analyzed MxA protein concentrations using the RDT MxA across 171 samples, with CHIKV and DENV RT-PCR serving as the reference standard. Based on RT-PCR results, samples were classified as CHIKV-positive (*n* = 20), DENV-positive (*n* = 67), or negative (*n* = 84). MxA concentrations differed significantly between groups (Kruskal–Wallis *H* = 32.35, *P* < 0.0001). Median MxA levels (IQR) were 29.94 ng/mL (10.24–51.84) for DENV-positive, 23.07 ng/mL (1.90–42.44) for CHIKV-positive, and 2.60 ng/mL (0–16.03) for PCR-negative samples.

**Fig 2:**
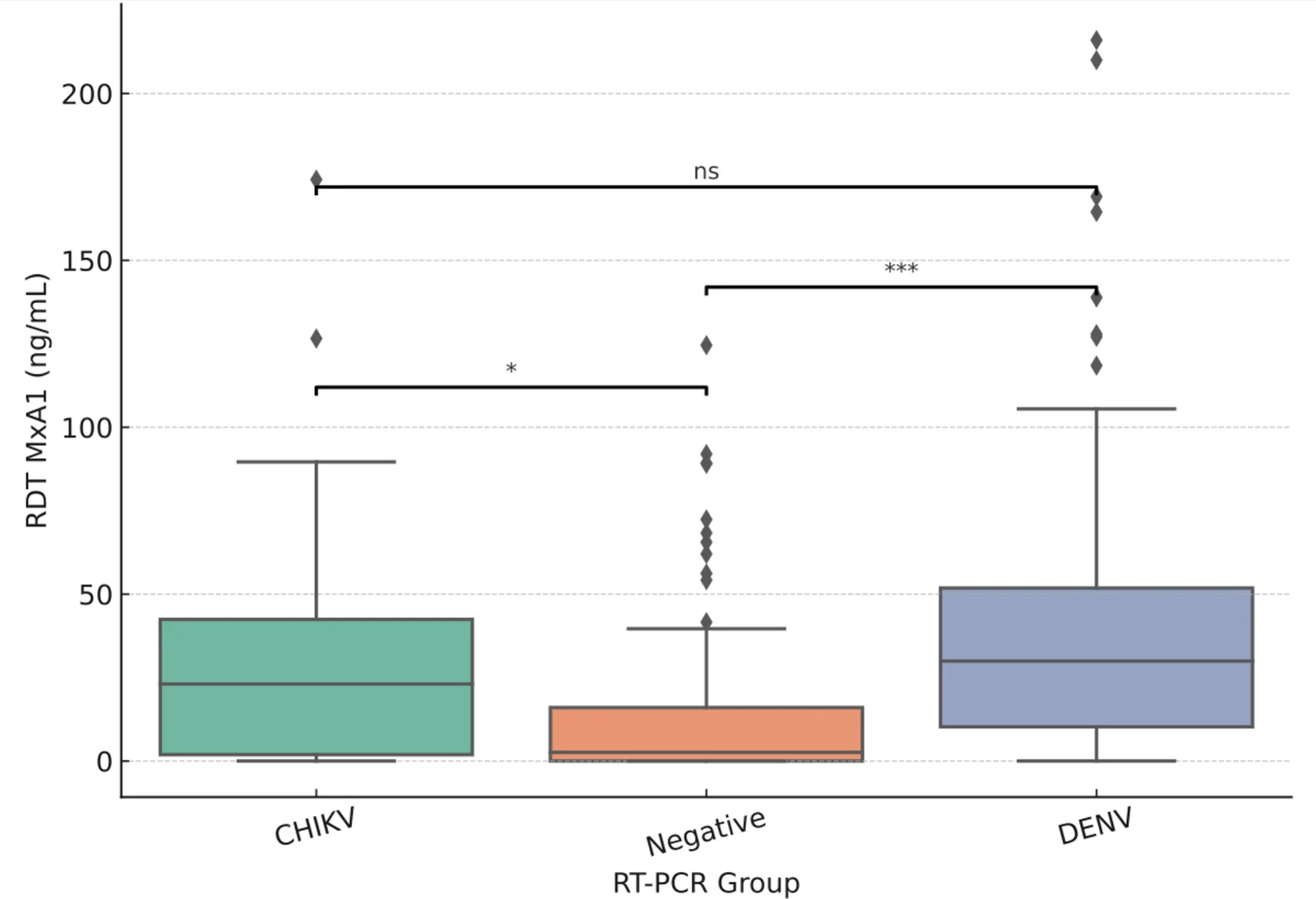
Distribution of MxA concentrations across diagnostic categories. MxA levels were significantly higher in DENV- and CHIKV-positive samples compared to RT-PCR–negative controls (Kruskal–Wallis *P* < 0.0001). No significant difference was observed between DENV and CHIKV groups. *P < 0.05, ***P < 0.001; ns, not significant.

Pairwise comparisons showed that both DENV- and CHIKV-positive groups had significantly higher MxA concentrations compared to negatives (*P* < 0.0001 and *P* = 0.0091, respectively), whereas no significant difference was observed between DENV- and CHIKV-positive groups (*P* = 0.323). These results confirm the expected interferon-driven MxA induction in acute arboviral infection and are summarized in Fig. 2.

### Diagnostic performance of MxA for viral infection

**Fig 3:**
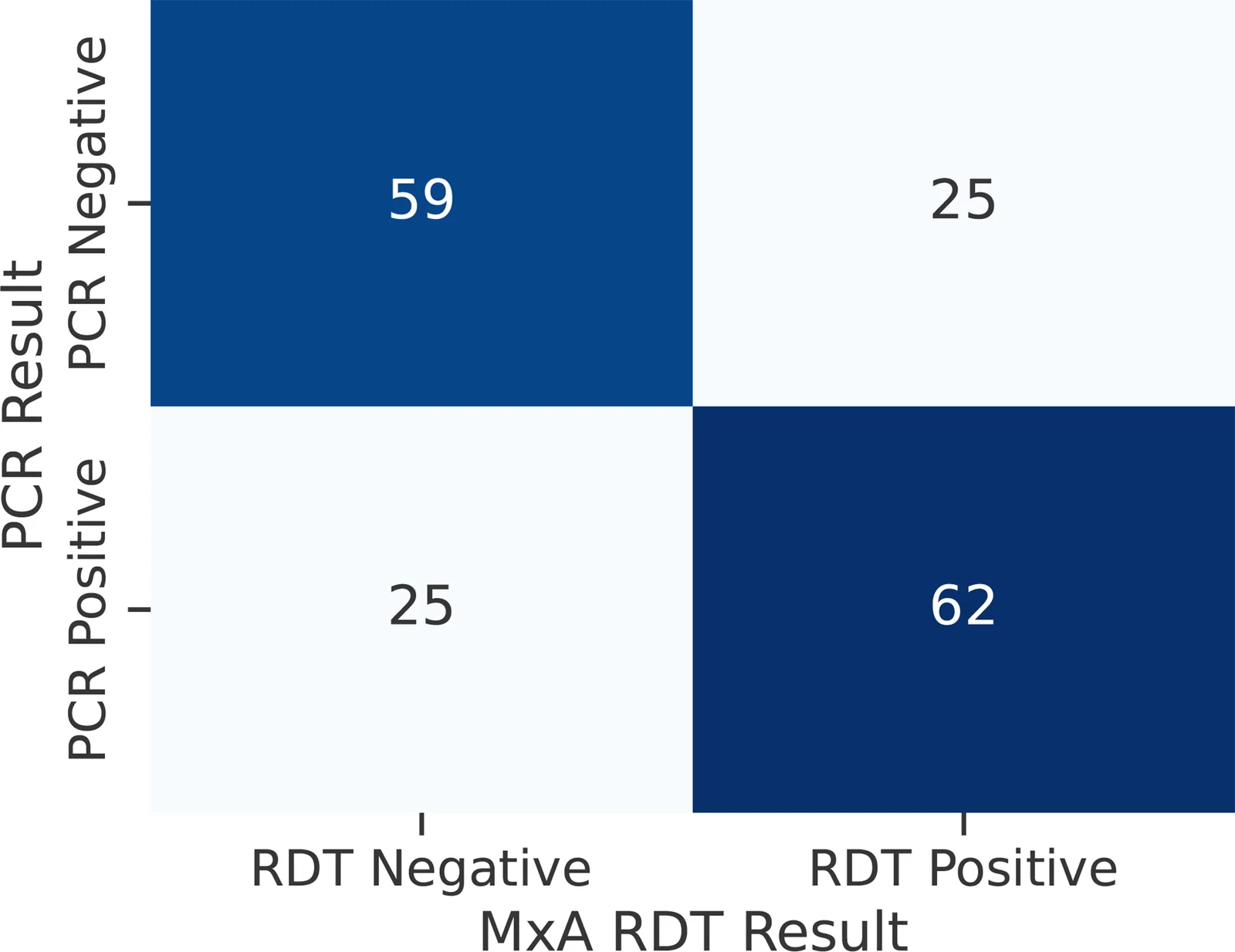
Confusion matrix showing diagnostic performance of MxA RDT vs RT-PCR. Using a positivity cut-off of 12 ng/mL, the RDT correctly identified 62/87 PCR-positive cases (sens. 71.3%) and 59/84 PCR-negative samples (spec. 70.2%), indicating moderate diagnostic accuracy.

To quantify diagnostic accuracy, we evaluated the performance of the MxA RDT against RT-PCR as the reference standard using a predefined positivity cut-off of 12 ng/mL. From the 171 samples, including 87 PCR-positive cases (67 DENV and 20 CHIKV) and 84 PCR-negative controls, the MxA RDT correctly identified 62/87 PCR-positive cases (Fig. 3), yielding a sensitivity of 71.3%, and classified 59/84 PCR-negative samples as negative, resulting in a specificity of 70.2%. Overall accuracy was 70.8%, PPV 71.3%, and NPV 70.2%. These results confirm the utility of MxA as a field-amenable host biomarker for detecting acute arboviral infections, with room for improvement in sensitivity and potential complementarity with pathogen-agnostic sequencing strategies.

**Fig 4:**
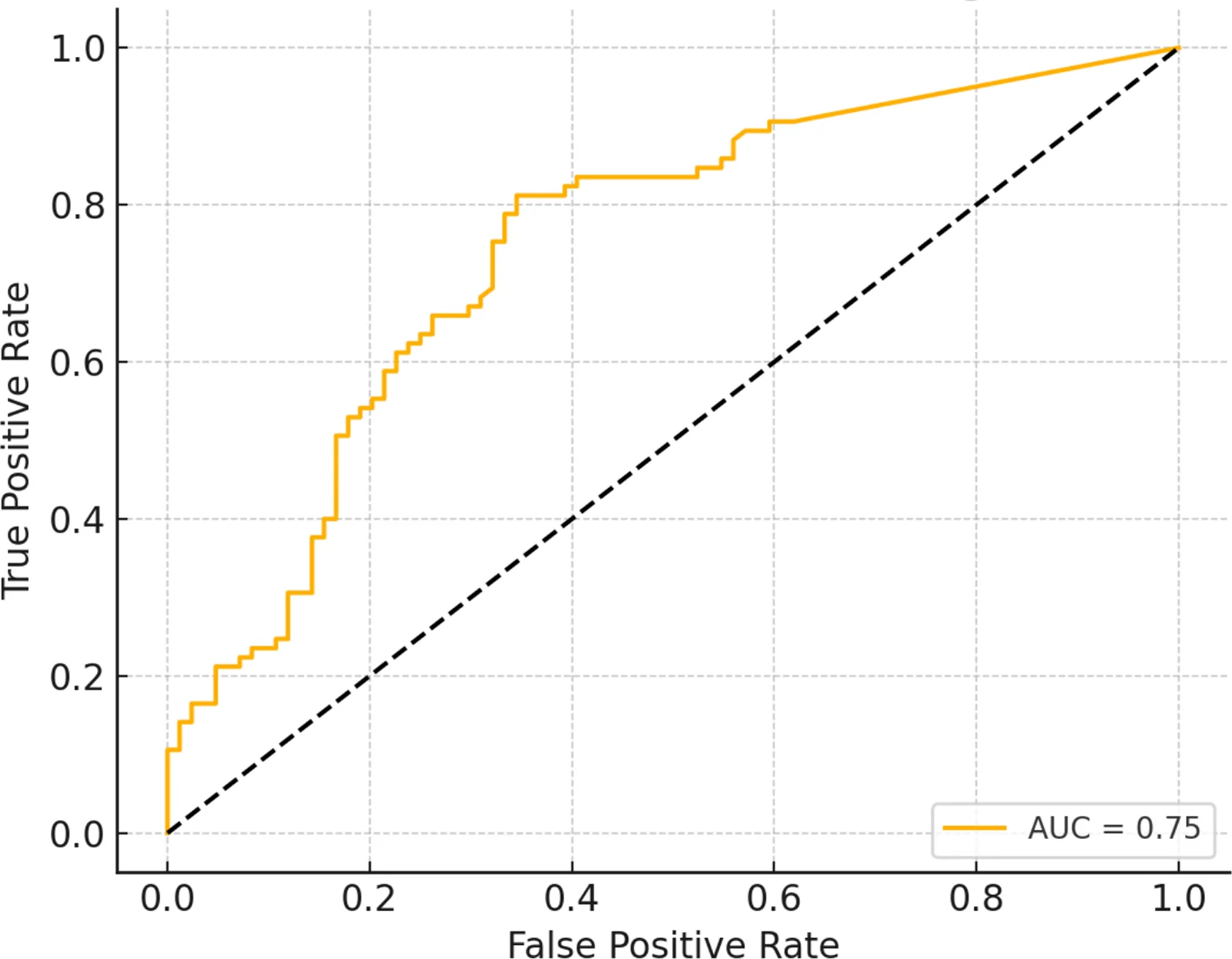
ROC curve for the MxA RDT in detecting arboviral infections. AUC = 0.75 (95% CI 0.68–0.82); sens. 71.3%, spec. 70.2% at 12 ng/mL.

ROC analysis confirmed the RDT’s moderate discriminatory power in this outbreak context, with an AUC of 0.75 (Fig. 4).

For the combined DENV/CHIKV classification using the manufacturer-recommended cut-off of 12 ng/mL, the AUC was 0.75 (95 % CI 0.68–0.82). At this cut-off, the RDT yielded a sensitivity of 71.3% (61.0–79.7), specificity of 70.2% (59.8–78.9), PPV of 71.3% (61.0–79.7), NPV of 70.2% (59.8–78.9), and an overall accuracy of 70.8% (63.5–77.1). The curve shape indicated a balanced trade-off between sensitivity and specificity at the chosen cut-off, supporting its field applicability in acute febrile illness triage.

**Fig 5:**
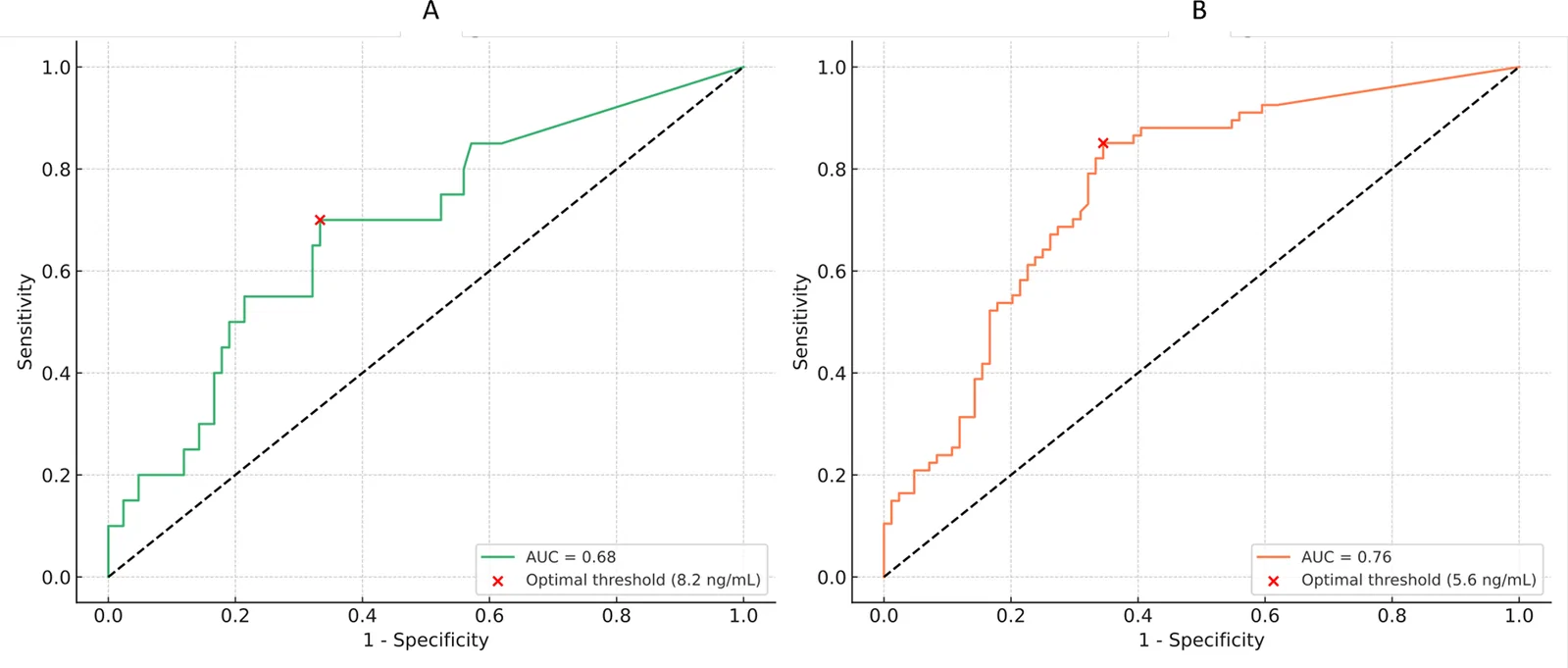
ROC curves stratified by virus type. (**A**) For DENV, AUC = 0.76 (optimal cut-off: 5.6 ng/mL). (**B**) For CHIKV, AUC = 0.68 (optimal cut-off: 8.2 ng/mL). DENV-associated MxA response was more predictive than CHIKV in this cohort.

To better understand the diagnostic performance of the MxA RDT in distinguishing individual arboviruses from non-viral febrile illness, we performed separate ROC analyses for DENV and CHIKV infections vs PCR-negative controls (Fig. 5).

For CHIKV, the AUC was 0.68 (95 % CI 0.58–0.78) at an optimal cut-off of 8.2 ng/mL, yielding sensitivity 70.0% (43.7–85.7), specificity 66.7% (56.4–76.7), PPV 33.3% (18.0–46.7), NPV 90.3% (81.2–96.4), and accuracy 67.3% (56.7–76.0). In comparison, the assay performed better for dengue, with an AUC of 0.76 (95 % CI 0.68–0.83) at a cut-off of 5.6 ng/mL, achieving sensitivity 85.1% (76.4–93.6), specificity 65.5% (54.5–74.7), PPV 66.3% (55.1–75.8), NPV 84.6% (74.1–91.8), and accuracy 74.2% (66.0–81.1).

These findings suggest differential host responses by virus type and highlight the potential for pathogen-specific optimization of host biomarker thresholds.

Because the same PCR-negative comparator group underpins all analyses, specificity remains stable across comparisons.

These results are summarized in Table 1.

**TABLE 1:** Diagnostic performance of MxA RDT for CHIKV and DENV infections using optimal thresholds, with bootstrapped 95% confidence intervals

Comparison	Threshold (ng/mL)	AUC	Sensitivity (95% CI)	Specificity (95% CI)	PPV (95% CI)	NPV (95% CI)	Accuracy (95% CI)
DENV + CHIKV vs negative	12^a^	0.75	71.3% (61%–79.7%)	70.2% (59.8%–78.9%)	71.3% (61%–79.7%)	70.2% (59.8%–78.9%)	70.8% (63.5%–77.1%)
DENV vs negative	5.6	0.76	85.1% (76.4%–93.6%)	65.5% (54.5%–74.7%)	66.3% (55.1%–75.8%)	84.6% (74.1%–91.8%)	74.2% (66%–81.1%)
CHIKV vs negative	8.2	0.68	70% (43.7%–85.7%)	66.7% (56.4%–76.7%)	33.3% (18%–46.7%)	90.3% (81.2%–96.4%)	67.3% (56.7%–76%)

^a^ Manufacturer-specified (IFU) cut-off.

### Assessment of malaria as a potential confounder

To further explore potential non-viral drivers of MxA positivity, we performed a pan-Plasmodium (malaria) conventional PCR (43) on a subset of 68 archived specimens with sufficient remaining sample volume for additional testing. Nineteen of 68 (27.9%) were malaria PCR positive. Within this subset, 7/68 specimens were CHIKV RT-qPCR positive, and 32/68 were DENV RT-qPCR positive. None of the malaria PCR-positive specimens were MxA positive by either RDT or ELISA, and none were DENV- or CHIKV-positive by RT-qPCR. Overall, these findings support no detectable association between malaria infection and MxA positivity in this retrospective data set. Nevertheless, because malaria testing was restricted to samples with adequate residual material, prospective studies with systematic parasitic and bacterial confirmation across the full panel will be important to further refine specificity estimates.

### Association between MxA levels and viral load categories

We analyzed MxA protein levels across semi-quantitative viral load categories, stratified by qPCR cycle threshold (Ct) values for CHIKV and DENV infections. Categories were defined using established RT-qPCR guidelines (44, 45): high (Ct < 25), medium (25 ≤ Ct < 30), low (30 ≤ Ct < 35), and very low or undetectable (Ct ≥ 35 or missing).

**Fig 6:**
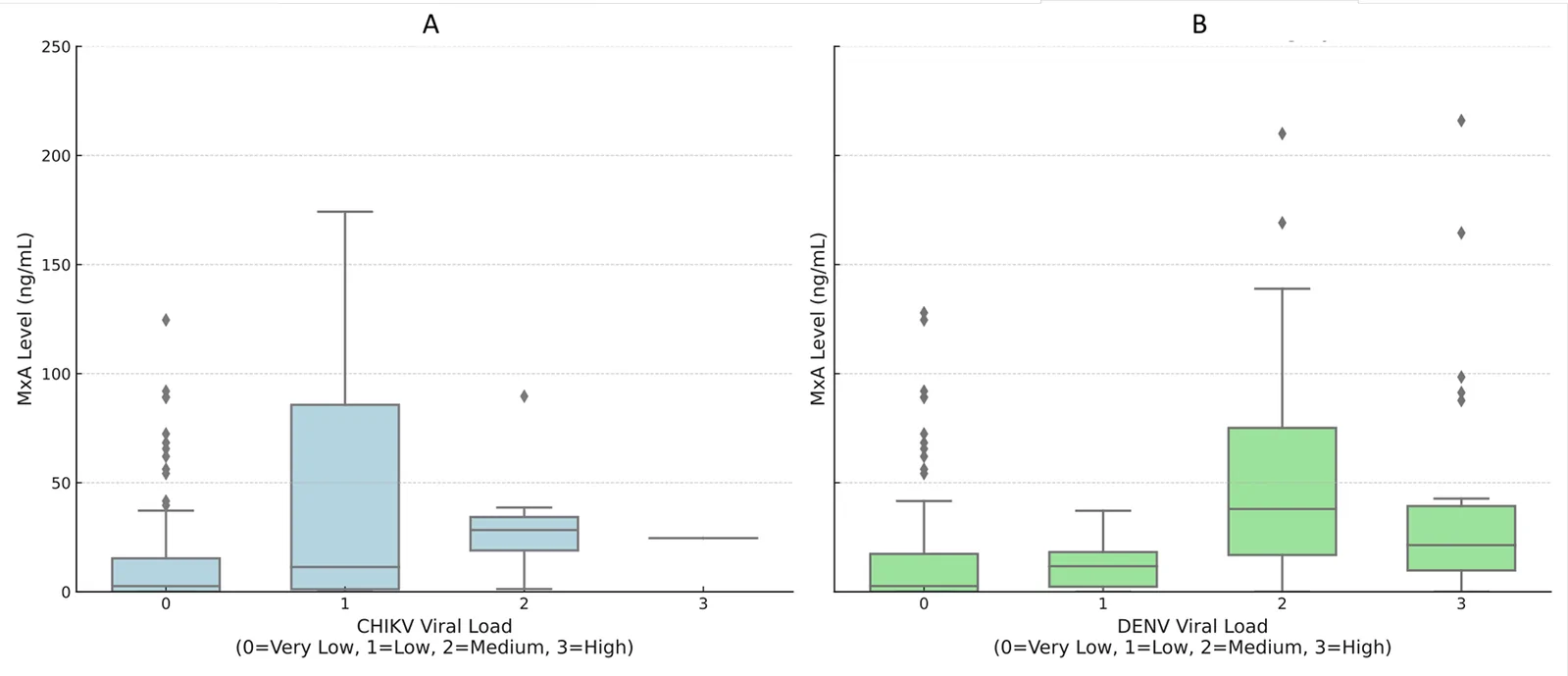
Association of MxA concentration with RT-qPCR viral load categories. (**A**) CHIKV samples showed increasing MxA with intermediate viral load. (**B**) DENV samples had significantly higher MxA levels in high/intermediate load groups (*P* < 0.0001).

For CHIKV (*n* = 20), the distribution was: high (*n* = 1), medium (*n* = 8), low (*n* = 9), and very low (*n* = 86). A significant difference in MxA levels was observed across categories (*H* = 9.42, *P* = 0.024). Mean MxA concentrations (ng/mL ± SD) were 24.6 (high, single sample), 31.5 ± 26.4 (medium), 50.6 ± 65 (low), and 14.6 ± 25.8 (very low). These trends are shown in Fig. 6A.

For DENV (*n* = 67), the sample distribution was high (*n* = 28), medium (*n* = 31), low (*n* = 7), and very low (*n* = 85). A highly significant difference in MxA levels was detected across groups (*H* = 33.37, *P* < 0.0001). Mean MxA concentrations were 38.9 ± 50.5 (high), 54.6 ± 52.7 (medium), 12.9 ± 13.2 (low), and 16.2 ± 28.7 (very low). This pattern is illustrated in Fig. 6B; Table S2.

These results reinforce the association between MxA expression and viral burden, particularly in the context of DENV infection, where higher MxA levels were generally observed in individuals with lower Ct values, reflecting higher viral loads. This pattern supports the biological rationale that MxA is upregulated in response to active viral replication and type I interferon signaling. While Ct-based binning provides a convenient and broadly applicableF proxy for estimating viral load, it is inherently semi-quantitative and influenced by assay conditions and sample quality. For more precise quantification of viral burden and its correlation with MxA expression, standardized methods using calibrated reference materials or digital PCR would be needed to enable absolute viral RNA quantification and inter-study comparability (41).

### Association of MxA levels with days since onset and age

We explored the relationship between MxA protein concentrations and two host-related variables: days since symptom onset and participant age. To focus on acute-phase dynamics, we excluded one sample with a reported onset of 57 days. The remaining 170 samples included 87 PCR-positive (DENV or CHIKV) and 83 PCR-negative cases.

When stratified by PCR status, no significant correlations were found between MxA levels and days since symptom onset. Among PCR-positive individuals, Spearman’s correlation coefficient was ρ = 0.073 (*P* = 0.501) and Pearson’s *r* = 0.024 (*P* = 0.828). Similarly, in the PCR-negative group, Spearman’s ρ = 0.095 (*P* = 0.401) and Pearson’s *r* = 0.021 (*P* = 0.853), indicating no meaningful linear or monotonic association (Table S3).

A similar lack of correlation was observed for participant age. In PCR-positive cases, Spearman’s ρ = 0.001 (*P* = 0.993) and Pearson’s *r* = 0.079 (*P* = 0.470). In PCR-negative individuals, Spearman’s ρ = –0.116 (*P* = 0.306) and Pearson’s *r* = –0.078 (*P* = 0.490).

**Fig 7:**
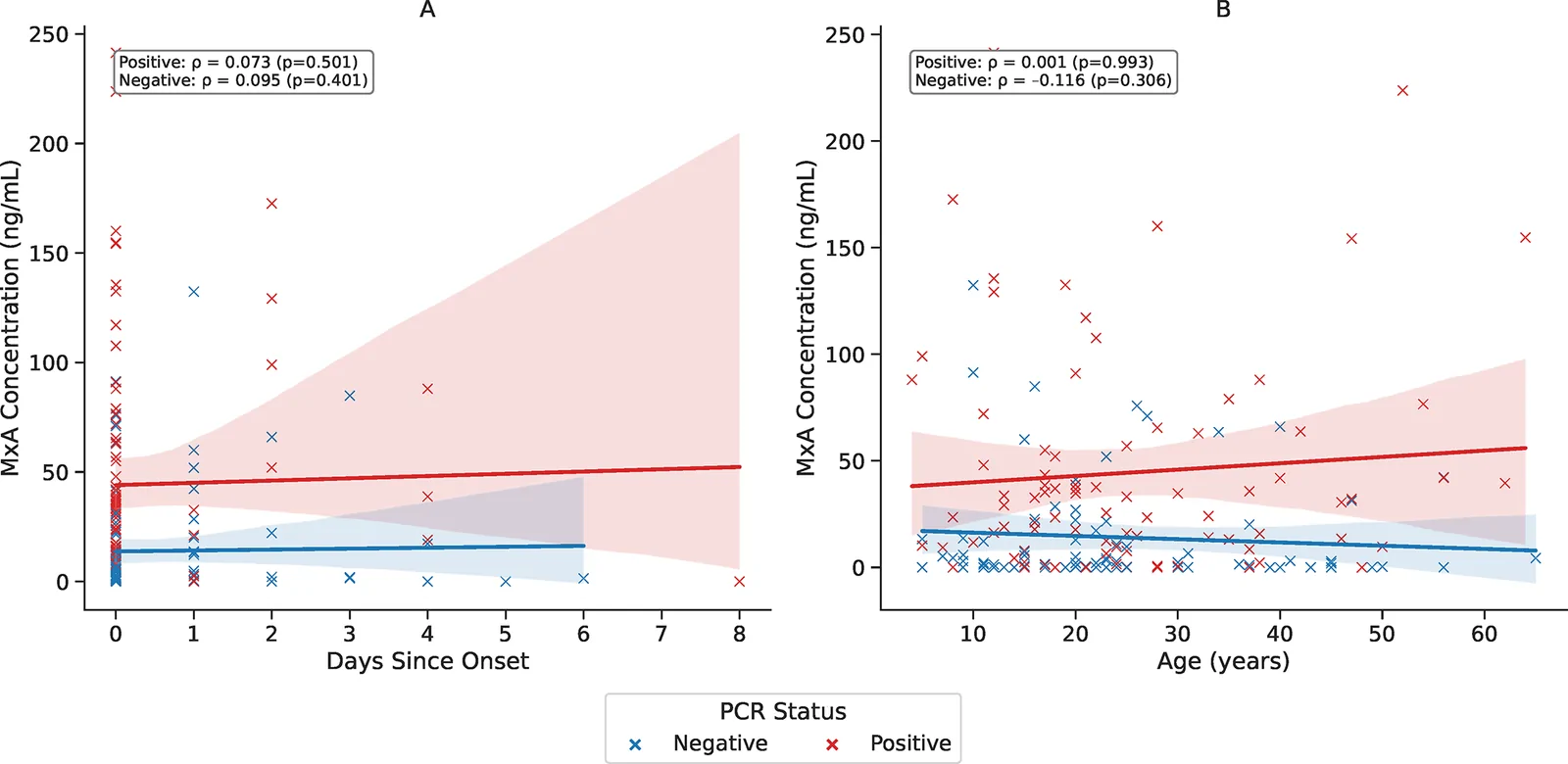
MxA expression relative to (**A**) days since symptom onset and (**B**) patient age. No significant correlations were observed between MxA concentration and either variable in both PCR-positive and -negative groups.

These results suggest that MxA expression in acute arboviral infection is independent of both symptom duration within the first 10 days and age of the patient. Regression plots with color-coded stratification by PCR status are shown in Fig. 7.

Given the non-pathogen-specific nature of MxA and the DENV/CHIKV-limited RT-qPCR reference, we used sequencing to better resolve a subset of discordant results.

### Sequencing of samples with discordant or unresolved diagnostic results

While DENV/CHIKV RT-qPCR served as the primary reference, given the outbreak context and prespecified targets, we performed a complementary sensitivity analysis using an expanded reference that incorporated sequencing adjudication of selected discordant samples. The sequencing workflow and findings used for this adjudication are described below.

### Arboviral infection identified by sequencing

To investigate potential missed infections, we performed high-throughput sequencing on samples that were positive by MxA RDT but negative by RT-qPCR for both DENV and CHIKV. A total of 25 serum samples were processed using two parallel approaches: unbiased metagenomic sequencing and targeted hybrid-capture using the Twist Comprehensive Viral Research Panel (Twist Biosciences), both on the Illumina platform. Each sample underwent both workflows, and concordant viral hits were observed across methods. This dual strategy increased detection confidence and ensured robustness of pathogen identification across viral families.

Among the sequenced samples, one was found to contain DENV-3, despite testing negative by RT-qPCR. This discordance likely reflects a viral load below the assay’s detection threshold rather than primer-template mismatches. DENV-3 was detected exclusively through the hybrid-capture sequencing approach, underscoring the enhanced sensitivity of targeted enrichment methods in low-titer infections. Phylogenetic analysis placed this DENV-3 sequence within genotype III, clustering tightly with strains circulating in Senegal since 2018, including isolates from recent national and regional outbreaks in Burkina Faso in 2023. This supports its classification as a locally acquired infection and highlights the role of sequencing in identifying circulating and epidemiologically relevant viral lineages (Fig. S2).

### Incidental viruses detected

Both metagenomic and hybrid-capture sequencing approaches identified incidental viral pathogens in two separate samples: Parvovirus B19 (B19V) and Torque teno virus (TTV). The TTV sequence grouped confidently within a major clade of human anelloviruses, consistent with the virus’s broad genetic diversity and common presence in human metagenomes (Fig. S3). For B19V, the phylogenetic tree confirmed its placement within genotype 1, validating its identity and underscoring the potential for such common viruses to emerge in unexpected clinical contexts (Fig. S4).

These viruses are not typically included in standard arboviral diagnostic panels, yet their identification highlights the broad pathogen-detection capability enabled by MxA pre-selection and untargeted sequencing. While TTV is generally considered non-pathogenic, its presence has been associated with immune activation and may serve as a marker of host immunological status. Importantly, this also underscores a limitation of MxA as a non-pathogen-specific marker of type I interferon activation: elevated MxA may occasionally reflect non-specific interferon responses and/or incidental viral carriage rather than the primary etiology of fever. In contrast, B19V is a clinically relevant pathogen linked to febrile exanthems, arthropathy, and transient aplastic crises, particularly in children and immunocompromised individuals. Its detection in our febrile patient cohort illustrates the added clinical value of a metagenomic approach for uncovering viral etiologies beyond those initially suspected. Further investigation, integrating prospective clinical adjudication, broader pathogen testing, and host-response profiling, will be important to resolve this diagnostic ambiguity in MxA-positive/arbovirus RT-qPCR–negative cases.

While these additional findings did not markedly alter the overall diagnostic performance estimates, they support the utility of broad-range sequencing as a powerful adjunct to refine pathogen detection and guide surveillance strategies in under-resourced settings.

## DISCUSSION

In this study, we evaluated the performance of an RDT and ELISA targeting MxA in the context of acute arboviral infections, specifically DENV and CHIKV, during recent outbreaks in Senegal. Our findings suggest that MxA could serve as a portable, host-response biomarker to support viral infection detection during arboviral outbreaks, particularly dengue. Importantly, the MxA-RDT showed moderate-to-substantial agreement with the MxA-ELISA when applying predefined manufacturer cut-offs (83.4% qualitative agreement; Cohen’s κ: 0.67), supporting the use of the RDT as a practical proxy for ELISA in field settings. Although the assay showed moderate sensitivity and specificity overall, performance varied by pathogen, and our study did not include bacterial infections for direct comparison. Given the malaria-endemic context, we additionally assessed malaria as a potential confounder in a subset analysis. However, the manufacturer reports that the RDT exhibits negligible induction by bacterial pathogens, based on prior evaluations. These findings highlight the potential of MxA to support clinical triage and help reduce unnecessary antibiotic use in settings where viral and bacterial fevers are difficult to distinguish at the bedside. Further validation, including studies with confirmed bacterial infections, is needed to fully establish its diagnostic value in real-world febrile illness management.

We observed that MxA levels were significantly higher in patients with PCR-confirmed arboviral infections than in negative controls, with median concentrations approximately 10 times greater in DENV and CHIKV cases. Both the RDT and ELISA showed consistent detection of these differences, confirming that MxA levels are robust and reproducible across platforms. These findings support the feasibility of using rapid MxA-based tools to detect the immune response to viral infections in settings where laboratory infrastructure is limited.

We found that overall MxA RDT sensitivity and specificity (71.3% and 70.2%, respectively) were moderate, but performance improved markedly for DENV when using a lower cut-off of 5.6 ng/mL compared to the combined DENV/CHIKV recommended cut-off (12 ng/mL).

By comparison, CHIKV required a higher cut-off of 8.2 ng/mL to optimize accuracy. This pathogen-specific variation likely reflects underlying differences in interferon-mediated MxA induction. CHIKV triggers strong immune responses involving type I interferons, which stimulate MxA production. DENV, by contrast, produces viral proteins that block interferon signals, possibly reducing MxA expression. This difference suggests that virus-specific cut-offs may be necessary to improve test performance, particularly when multiple viruses are circulating.

We also examined how MxA levels relate to patient characteristics. We found no clear association between MxA levels and patient age or number of days since symptom onset, indicating that MxA remains a stable early marker during the acute phase of illness. However, we found that higher MxA levels were associated with lower RT-qPCR Ct values, reflecting higher viral load. This supports the biological link between MxA and active viral replication.

Despite moderate accuracy (~70%), the ease of use, affordability, and rapidity of the MxA RDT still make it highly valuable as a frontline triage tool in resource-limited settings, where the alternative is often no immediate diagnostic information. In this context, MxA should be viewed primarily as a prioritization signal rather than a stand-alone etiological diagnosis: a positive result can support early referral, confirmatory RT-qPCR, and/or targeted sequencing, whereas a negative result does not exclude viral infection and should be interpreted alongside clinical assessment and local epidemiology. Operationally, deploying MxA at first contact could help streamline patient pathways, reduce unnecessary empiric antibiotics when bacterial disease is unlikely, and focus scarce molecular capacity on those most likely to benefit from confirmatory testing during outbreaks.

Nevertheless, the moderate standalone accuracy also highlights the need to move toward multiplex host-response panels to improve performance across heterogeneous febrile syndromes. Combining an interferon-driven marker (MxA) with inflammation-associated markers like CRP or PCT and other immune signatures could better separate viral, bacterial, and mixed infections and may be particularly valuable in co-infections or in patients with altered immune responses (e.g., immunosuppression, chronic infections, and extremes of age), where interferon induction may be blunted or non-specific. Prospective evaluations should therefore assess multiplex algorithms, define context-specific cutoffs, and quantify added value over MxA alone in real-world febrile illness workflows.

A major contribution of our study is the use of genomic sequencing to investigate cases in which MxA levels were high, but PCR tests were negative. Through unbiased metagenomics and targeted capture, we detected other viruses such as DENV-3, human erythrovirus V9, and torque teno virus. These findings underscore critical diagnostic blind spots in routine surveillance, which often rely on preselected panels and may miss unexpected, emerging, or divergent pathogens. At the same time, the detection of highly prevalent viruses such as TTV underscores that MxA reflects type I interferon activation rather than pathogen specificity, and elevated MxA may occasionally coincide with incidental viral carriage or immune activation unrelated to the primary febrile etiology. This diagnostic ambiguity supports the need for integrated interpretation combining sequencing results with clinical correlation and, where feasible, broader pathogen testing and host-response profiling (46–48).

The value of a broad, agnostic approach to pathogen detection is further emphasized by past outbreak experiences. For instance, during the 2014–2015 Ebola virus disease outbreak in Guinea, surveillance efforts heavily focused on Ebola overlooked the emergence and circulation of vaccine-derived polioviruses until several paralytic cases occurred (49). This illustrates how diagnostic blind spots arise during complex emergencies, underscoring the need for integrated approaches that combine host biomarkers with metagenomic sequencing to flag unexpected viral threats early.

While MxA has been widely studied in respiratory infections, our findings extend its utility to arboviral diseases, a major yet underdiagnosed burden in tropical regions. The ability of MxA to act as a general interferon-stimulated gene marker suggests its broader utility where pathogen-specific diagnostics are not readily available.

Building on respiratory settings, where FebriDx, a rapid finger-stick assay measuring MxA and CRP, routinely achieved >85% sensitivity for bacterial vs non-bacterial illness and 88%–94% specificity for viral infection (50, 51), we show that the same host response signature applies during dengue and chikungunya outbreaks in Senegal. In our cohort, median MxA concentrations were 10 times higher in PCR-confirmed arboviral cases than in controls, and both rapid test and ELISA formats captured these elevations reliably.

Moreover, just as respiratory studies optimized combined CRP–MxA cut-offs, we found that virus-specific MxA thresholds increased sensitivity for dengue from 66% to over 85%. This demonstrates that, despite divergent viral immune evasion strategies—DENV partial interferon antagonism vs CHIKV robust IFN induction—MxA remains a dependable proxy for active viral replication.

Finally, sequencing of MxA-positive but RT-PCR-negative samples uncovered additional pathogens—DENV-3 and B19V genotype 1—each in a distinct patient, that routine panels missed. This mirrors respiratory findings where elevated MxA flagged clinically significant infections despite negative PCR (52). Together, our work confirms MxA versatility beyond respiratory disease and highlights its promise for rapid triage, targeted sequencing, and improved outbreak surveillance in resource-limited, arbovirus-endemic settings. The detection of B19V in an MxA-positive, PCR-negative patient underscores the diagnostic value of sequencing in resolving febrile illness of unknown etiology. Although often asymptomatic, B19V can cause acute symptoms, such as rash, fever, and arthralgia, that closely resemble arboviral syndromes. In this context, B19V may represent a true etiological agent rather than an incidental finding or bystander. This highlights the importance of incorporating broad-range sequencing to differentiate clinically relevant infections from viral “noise” in high-background settings.

In contrast, the detection of TTV in another MxA-positive, RT-PCR–negative sample is more consistent with incidental anellovirus carriage and/or non-specific interferon activation than with an acute etiologic role. TTV is a highly prevalent, non-enveloped DNA virus that establishes chronic, typically asymptomatic infections and is not known to cause acute febrile illness. Its detection, particularly in low titer or partial genome fragments, is commonly interpreted as a surrogate marker of host immune status rather than direct pathogenicity. The presence of TTV in this case may therefore represent persistent viral antigenemia or low-level replication triggering innate immune responses, captured by elevated MxA levels. This underscores a key limitation of host-response biomarkers such as MxA: while highly sensitive to interferon activity, they may not always indicate clinically relevant or causative infections, especially in populations with high background viral burden or altered immune states. Differentiating between true pathogen-driven interferon responses and non-specific immune activation remains a major challenge for host-based diagnostics. Future studies integrating MxA with host-response profiling (e.g., targeted transcript signatures and inflammatory markers), quantitative viral load measurements, and clinical correlation will be essential to resolve this diagnostic gray zone.

Our study has limitations. It utilized retrospective samples from outbreak investigations, which may not fully represent real-time clinical care scenarios. Additionally, although we included PCR-negative febrile controls, these samples were not systematically confirmed as bacterial or parasitic infections, which may limit the rigor of specificity estimates.

MxA is a non-pathogen-specific marker of type I interferon activation (5) and can therefore be induced by infections beyond DENV/CHIKV (and, less commonly, by non-infectious inflammatory states). Consequently, when DENV/CHIKV RT-qPCR is the primary reference standard, some MxA-positive results may reflect alternative etiologies and be counted as “false positives,” reducing apparent specificity. To partially address potential parasitic confounding in this malaria-endemic setting, we performed pan-Plasmodium PCR on a subset of residual specimens with sufficient remaining volume; malaria positivity was not associated with MxA positivity in this subset. Nevertheless, this analysis was limited to available residual material, and prospective studies that include systematically confirmed bacterial and parasitic infections as controls are needed to more rigorously assess diagnostic specificity and clinical utility. Larger prospective, multicenter evaluations will further validate these findings across broader patient populations and etiologic spectra.

From a public health perspective, MxA could significantly enhance syndromic surveillance strategies. In areas with delayed pathogen-specific tests, general viral biomarkers like MxA could trigger early outbreak responses. Additionally, MxA RDTs’ portability, simplicity, and minimal infrastructure needs make them ideal for peripheral health centers and mobile units in resource-limited environments.

Future prospective studies should evaluate context-adapted multiplex host-response algorithms and broader etiologic panels and validate performance across co-infections and immunologically heterogeneous patient groups. Studies should also extend the pathogen range beyond DENV and CHIKV to include other medically significant arboviruses (e.g., Zika virus, yellow fever virus, and Rift Valley fever virus) and hemorrhagic fever viruses (e.g., Ebola and Lassa). This broader evaluation is essential to define MxA diagnostics’ full scope and specificity.

Future research should also include health economic analyses comparing the cost-effectiveness of implementing rapid MxA-based diagnostics vs standard molecular tests, as this could greatly influence policy adoption and scalability in low-resource settings (53). Efforts to scale up MxA diagnostics must also address access equity, procurement, and supply-chain stability (forecasting, stock management, lot traceability, and storage requirements), and integration into existing surveillance platforms in low- and middle-income countries.

Successful deployment will also require standardized operator training and competency, as well as feasible quality assurance/quality control procedures (e.g., lot verification on receipt, reader verification/calibration where applicable, documentation of invalid results, and repeat/duplicate testing or escalation for borderline or discordant results). Implementation will benefit from simple result capture and reporting (paper or digital) linked to fever algorithms, specimen referral networks, and downstream confirmatory testing. Comparative evaluations of MxA alongside widely used markers such as CRP and PCT could clarify its relative strengths in viral detection. Clear regulatory pathways and diagnostic validation protocols will be essential to support rapid approval and deployment of MxA RDTs, including context-appropriate performance targets and post-market monitoring where feasible.

These points align with WHO’s call for innovative diagnostics that are affordable, scalable, and adaptable to evolving outbreak threats (54). Given the portability of MxA RDTs, integration with digital health systems for real-time reporting could significantly enhance early detection and response capacities, particularly in remote or outbreak-prone regions. Finally, extending host-response biomarker approaches to animal or environmental surveillance might facilitate earlier detection of zoonotic spillovers before human cases emerge.

In summary, this study provides the first field-based validation of an MxA rapid diagnostic test during arboviral outbreaks in West Africa. We demonstrate that MxA testing can be feasibly deployed and adds diagnostic value when combined with genomic tools. MxA-based diagnostics could become a vital component of outbreak preparedness by enabling early detection of viral illness even when specific tests are unavailable. A prospective implementation study is warranted to assess clinical impact, cost-effectiveness, and the integration of MxA testing into routine care and surveillance pathways.

## Supplementary Material

Reviewer comments

## SUPPLEMENTAL MATERIAL

Figure S1 spectrum.03392-25-s0001.pdf Simplified study workflow integrating routine DENV/CHIKV RT-qPCR diagnostics, MxA testing, and sequencing of discordant cases.

Figure S2 spectrum.03392-25-s0002.pdf Phylogenetic analysis of dengue virus sequence identified in this study.

Figure S3 spectrum.03392-25-s0003.pdf Phylogenetic tree of TTV identified by metagenomic sequencing.

Figure S4 spectrum.03392-25-s0004.pdf Phylogenetic placement of parvovirus B19 sequence identified in this study.

Legends spectrum.03392-25-s0005.docx Legends for Fig. S1 to S4.

Table S1 spectrum.03392-25-s0006.xlsx Clinical and demographic characteristics of study participants.

Table S2 spectrum.03392-25-s0007.xlsx Summary statistics of MxA protein concentrations stratified by virus type and viral load category.

Table S3 spectrum.03392-25-s0008.xlsx Spearman and Pearson correlation coefficients examining the relationship between MxA protein levels and days since symptom onset or patient age, stratified by PCR status.

OPEN PEER REVIEW reviewer-comments.pdf An accounting of the reviewer comments and feedback.

ASM does not own the copyrights to Supplemental Material that may be linked to, or accessed through, an article. The authors have granted ASM a non-exclusive, world-wide license to publish the Supplemental Material files. Please contact the corresponding author directly for reuse.
