# Supplementary material for: Evaluating myxovirus resistance protein A-based rapid testing combined with pathogen sequencing for arboviral and incidental viral infection surveillance in Senegal
Source: Microbiol Spectr. 2026 Jun 3;14(7):e03392-25. doi: 10.1128/spectrum.03392-25 (PMC13340290; doi:10.1128/spectrum.03392-25)

Simplified workflow: routine RT-qPCR → archived panels → MxA testing → sequencing of discordant cases

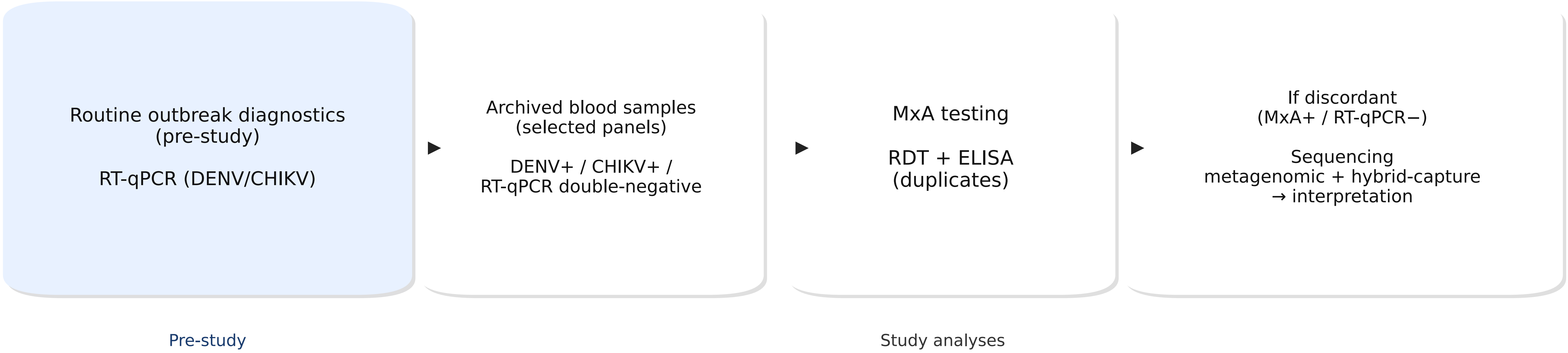

Supplement: Figure S1 — Simplified study workflow integrating routine DENV/CHIKV RT-qPCR diagnostics, MxA testing, and sequencing of discordant cases. [file spectrum.03392-25-s0001.pdf]
