# Supplementary figures and images for: Evaluating myxovirus resistance protein A-based rapid testing combined with pathogen sequencing for arboviral and incidental viral infection surveillance in Senegal
Source: Microbiol Spectr. 2026 Jun 3;14(7):e03392-25. doi: 10.1128/spectrum.03392-25 (PMC13340290; doi:10.1128/spectrum.03392-25)

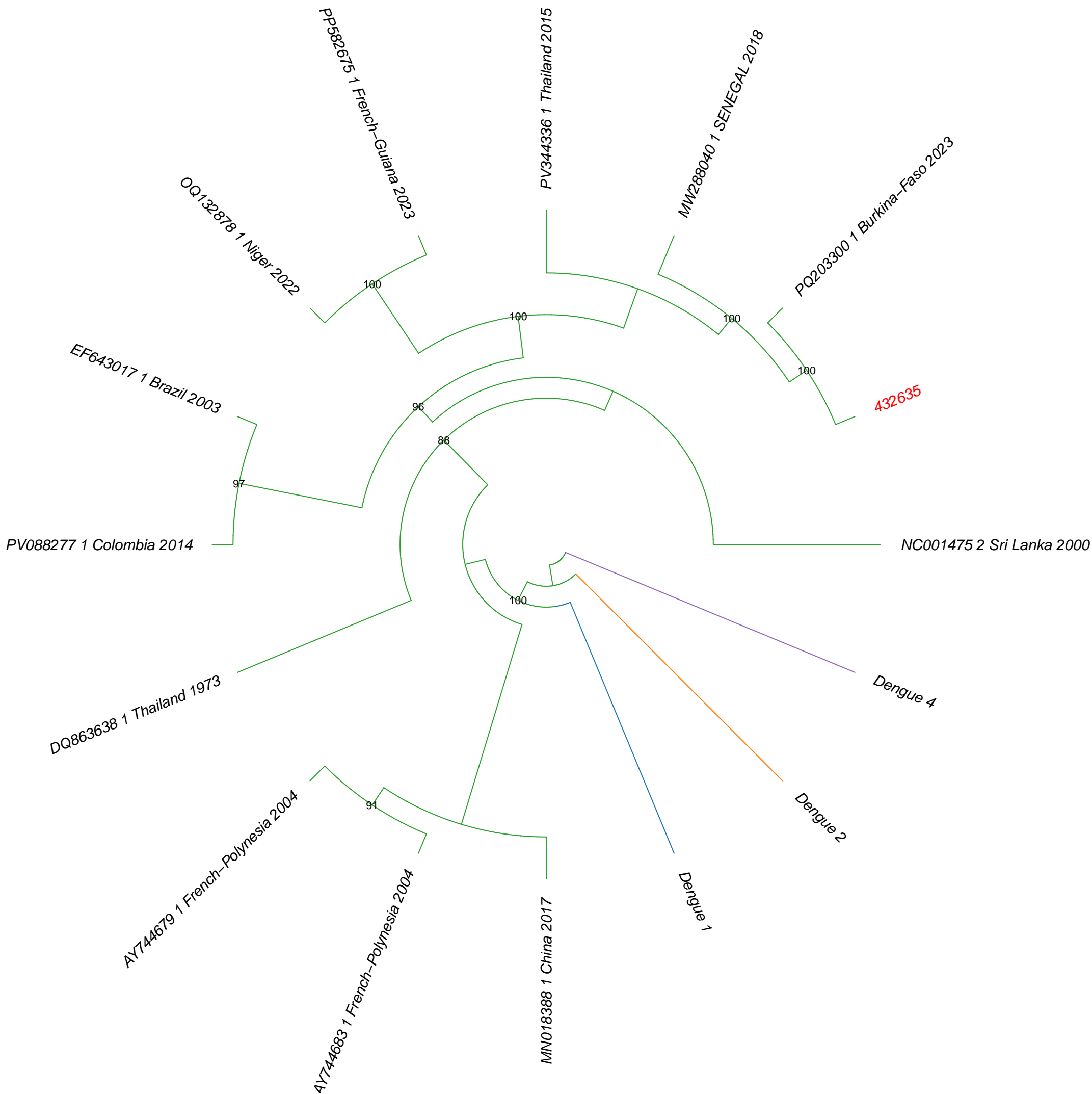

Supplement: Figure S2 — Phylogenetic analysis of dengue virus sequence identified in this study. [file spectrum.03392-25-s0002.pdf]

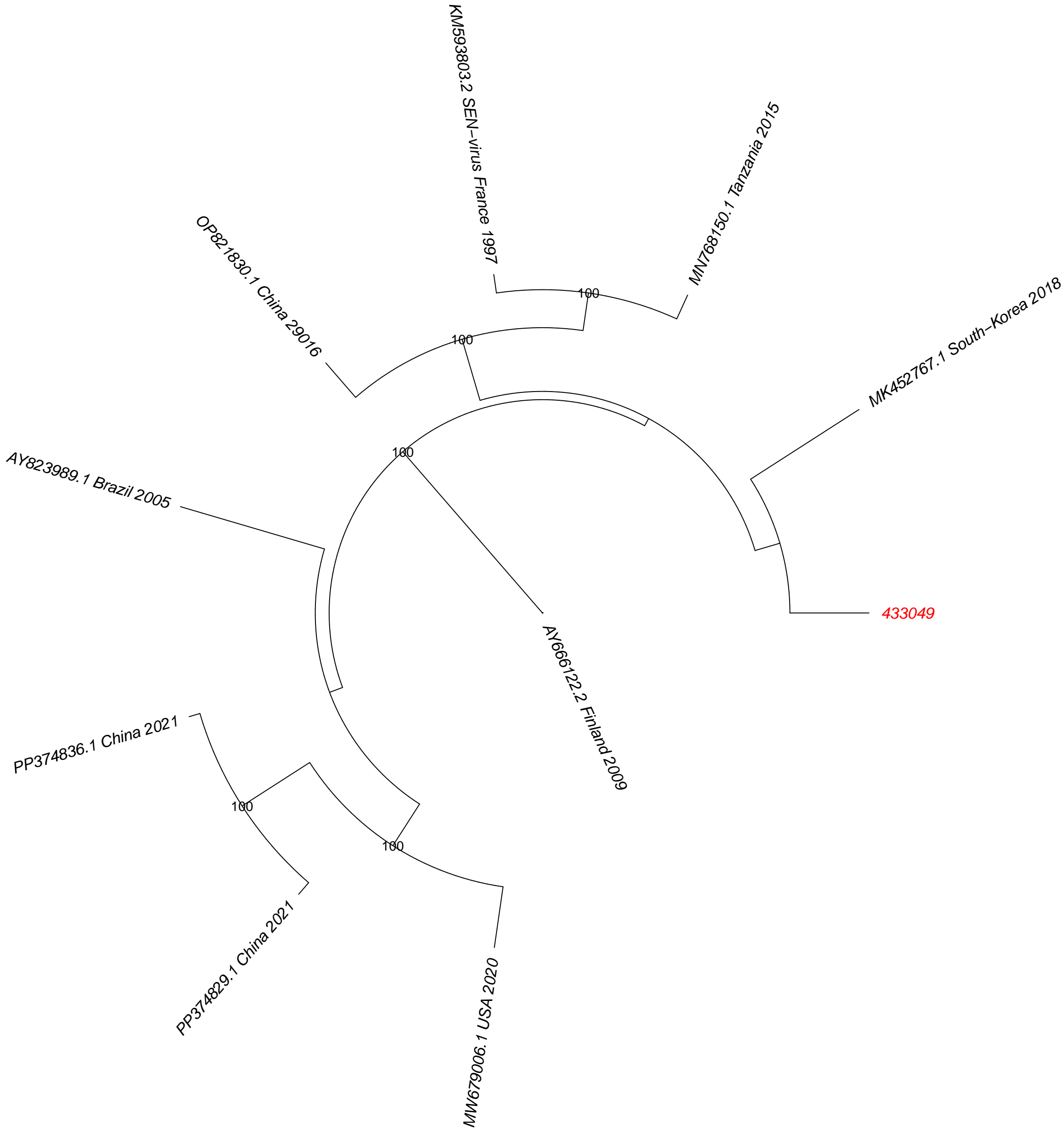

Supplement: Figure S3 — Phylogenetic tree of TTV identified by metagenomic sequencing. [file spectrum.03392-25-s0003.pdf]

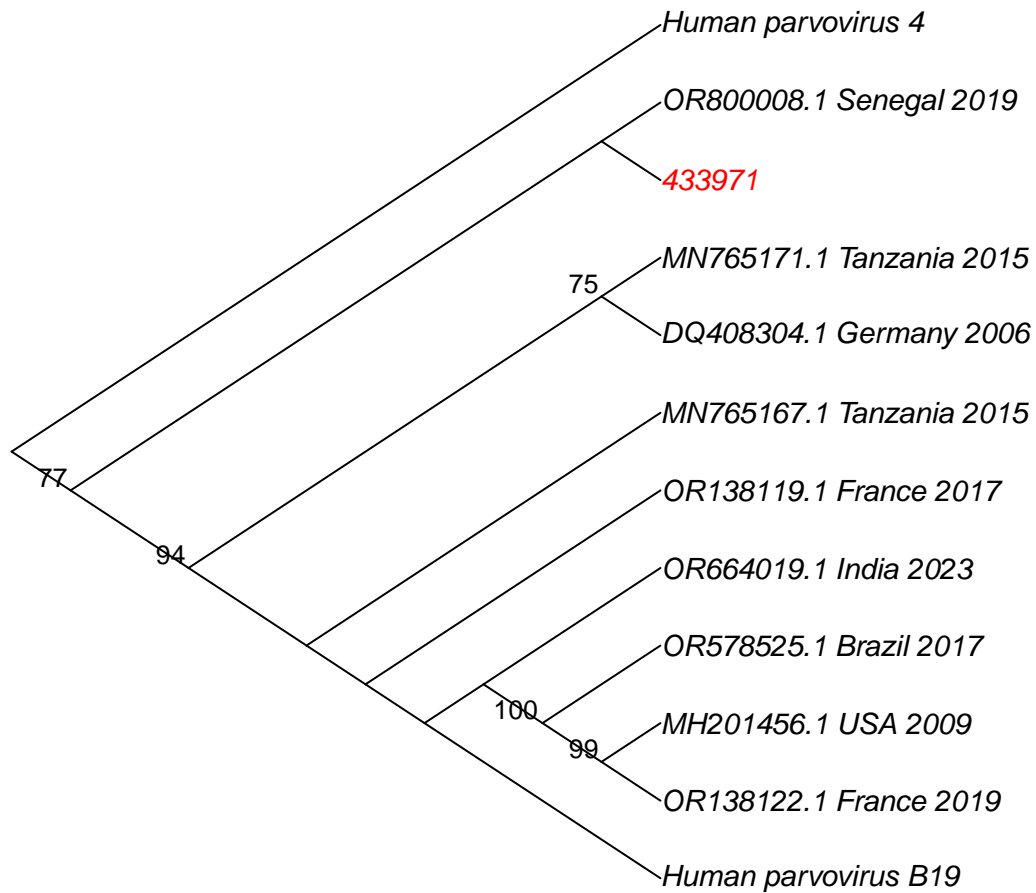

Supplement: Figure S4 — Phylogenetic placement of parvovirus B19 sequence identified in this study. [file spectrum.03392-25-s0004.pdf]
