# Supplementary material for: Evaluating myxovirus resistance protein A-based rapid testing combined with pathogen sequencing for arboviral and incidental viral infection surveillance in Senegal
Source: Microbiol Spectr. 2026 Jun 3;14(7):e03392-25. doi: 10.1128/spectrum.03392-25 (PMC13340290; doi:10.1128/spectrum.03392-25)
Supplement: Legends — for Fig. S1 to S4. [file spectrum.03392-25-s0005.docx]

Fig S1: Simplified study workflow integrating routine DENV/CHIKV RT-qPCR diagnostics, MxA testing, and sequencing of discordant cases.

Fig S2: Phylogenetic analysis of dengue virus sequence identified in this study. Maximum-likelihood tree constructed using nealy complete sequences of dengue virus (DENV), including representatives of all four DENV serotypes. The DENV-3 strain identified in this study is highlighted and clusters within DENV-3-genotype III strains, including recent isolates from Burkina Faso 2023. The tree is rooted using a DENV-4 sequence to illustrate phylogenetic relationships across serotypes.

Fig S3: Phylogenetic tree of Torque teno virus (TTV) identified by metagenomic sequencing. Maximum-likelihood tree showing partial sequences ((~2,200 nt) of TTV, along with the sequence identified in this study. The tree is rooted to indicate directionality; all terminal nodes correspond to TTV sequences.

Fig S4: Phylogenetic placement of parvovirus B19 sequence identified in this study. Maximum-likelihood tree of representative human parvovirus B19 (B19V) sequences. The nearly complete genome identified in this study clusters within genotype 1, the most common circulating lineage worldwide. Human parvovirus 4 was included as an outgroup to root the tree and clarify the phylogenetic relationships among B19V genotypes.
