# Supplementary material for: Evaluating myxovirus resistance protein A-based rapid testing combined with pathogen sequencing for arboviral and incidental viral infection surveillance in Senegal
Source: Microbiol Spectr. 2026 Jun 3;14(7):e03392-25. doi: 10.1128/spectrum.03392-25 (PMC13340290; doi:10.1128/spectrum.03392-25)
Supplement: Reviewer comments [file reviewer-comments.pdf]

AMERICAN SOCIETY FOR MICROBIOLOGY | Microbiology Spectrum

Evaluating Myxovirus Resistance Protein A-Based Rapid Testing Combined with Pathogen Sequencing for Arboviral and Incidental Viral Infection Surveillance in Senegal.

Mouhamed Kane, Serge Moukaha Doukanda, Safietou SANKHE, Bocar Sow, Marie Ndione, Moundhir Mhamadi, Madeleine Dieng, Seynabou Diop, Seynabou Seye, Maimouna MBANNE, Oumar Faye, Mamadou Barry, Mbacké SEMBENE, Cheikh Loucoubar, Gamou Fall, Amadou Diallo, Cheikh DIAGNE, Ndongo Dia, and Moussa DIAGNE

Corresponding Author(s): Moussa DIAGNE, Institut Pasteur de Dakar

Review Timeline:

Submission Date:	October 23, 2025
Editorial Decision:	January 14, 2026
Revision Received:	February 14, 2026
Accepted:	March 10, 2026

Editor: Weiqiang Chen

Reviewer(s): Disclosure of reviewer identity is with reference to reviewer comments included in decision letter(s). The following individuals involved in review of your submission have agreed to reveal their identity: Desalegn Abebaw (Reviewer #2)

Transaction Report:

(Note: With the exception of the correction of typographical or spelling errors that could be a source of ambiguity, letters and reports are not edited. The original formatting of letters and referee reports may not be reflected in this compilation.)

DOI: https://doi.org/10.1128/spectrum.03392-25

Re: Spectrum03392-25 (**Evaluating Myxovirus Resistance Protein A-Based Rapid Testing Combined with Pathogen Sequencing for Arboviral and Incidental Viral Infection Surveillance in Senegal.**)

Dear Dr. Moussa Moise DIAGNE:

Thank you for the privilege of reviewing your work. Below you will find my comments, instructions from the Spectrum editorial office, and the reviewer comments.

Reviewer 1:

General comments
This study presents an important field evaluation of MxA-based rapid diagnostic tests (RDT) and ELISA in detecting acute arboviral infections, particularly dengue virus (DENV) and chikungunya virus (CHIKV), in outbreak settings in Senegal. The integration of host-response biomarker analysis with genomic sequencing to identify additional viral pathogens is a notable strength, providing valuable insight into diagnostic blind spots. The findings highlight the potential role of MxA as a rapid, portable, and affordable biomarker to support clinical triage and outbreak surveillance in resource-limited environments. The manuscript is generally well written, logically structured, and makes a convincing case for further development and validation of MxA diagnostics in tropical arbovirus-endemic regions.

Major comments
1. The retrospective design and use of outbreak samples limit the generalizability to routine clinical care. Patient selection criteria and relevant clinical metadata are not fully described, which may introduce bias and confounders affecting MxA levels. Prospective studies including systematically confirmed bacterial and parasitic infections as controls are needed to rigorously assess diagnostic specificity.
2. The reported moderate sensitivity and specificity (~70%) of the MxA RDT indicate promise but are below ideal diagnostic thresholds. The virus-specific thresholds are a useful step; however, the manuscript should discuss more clearly the clinical implications of this moderate accuracy and the need for multiplex biomarker panels to improve performance, especially in co-infections or immunocompromised patients.
3. While the use of genomic sequencing to identify missed viral pathogens in MxA-positive but RT-PCR-negative samples is a strong point, the detection of Torque teno virus underscores a limitation: elevated MxA may reflect nonspecific interferon responses unrelated to acute infection. This diagnostic ambiguity requires further investigation through additional molecular profiling and clinical correlation.
4. The discussion would benefit from deeper consideration of practical challenges for implementing MxA diagnostics in low-resource settings, including supply chain logistics, training, quality control, and integration into existing health and surveillance infrastructures, to better inform real-world applicability.

Minor comments
1. The methods section should clarify sample storage conditions and steps taken to minimize assay variability or batch effects.
2. Adding a schematic figure illustrating the workflow combining MxA testing with metagenomic sequencing could improve reader understanding.
3. Please clarify if MxA testing was performed blinded to RT-PCR and sequencing results to avoid bias.
4. Consistency in terminology between "cut-off" and "threshold" is recommended for clarity; minor typographical and grammatical errors should be addressed.
5. A brief discussion on potential MxA induction in non-viral inflammatory conditions and its impact on specificity would strengthen the manuscript.

Reviewer 2:
This manuscript evaluates the performance of MxA-based RDT, combined with pathogen sequencing, an approach highly relevant in resource-limited settings with frequent diagnostic uncertainty. The authors also explore other incidental causes in MxA-positive samples. While the study is of interest, a few concerns should be addressed.
1. In this manuscript, the authors are expected to report the level of agreement between MXA-RDT and MXA-ELISA using kappa statistics based on predefined cutoff values.
2. In subsection 4.3 of the Results, the non-specific nature of MxA may lead to an overestimation of positivity in MxA RDT when only DENV and CHIKV PCR are used as references. The authors may want to justify this approach and assess MxA RDT performance using combined PCR and sequencing data as a reference.
3. Typographic errors should be corrected-for example, the initials of the paragraph in section 4.6.2 (lines 423 and 424).
4. The source (Word file) does not have line numbers

Please return the manuscript within 60 days; if you cannot complete the modification within this time period, please contact me. If you do not wish to modify the manuscript and prefer to submit it to another journal, notify me immediately so that the manuscript may be formally withdrawn from consideration by Spectrum.

Revision Guidelines
To submit your modified manuscript, log into the submission site at https://spectrum.msubmit.net/cgi-bin/main.plex. Go to Author Tasks and click the appropriate manuscript title to begin. The information you entered when you first submitted the paper will be displayed; update this as necessary. Note the following requirements:

• Upload point-by-point responses to the issues raised by the reviewers in a file named "Response to Reviewers," NOT in your cover letter.
• Upload a compare copy of the manuscript (without figures) as a "Marked-Up Manuscript" file.
• Upload a clean .DOC/.DOCX version of the revised manuscript and remove the previous version.
• Each figure must be uploaded as a separate, editable, high-resolution file (TIFF or EPS preferred), and any multipanel figures must be assembled into one file.
• Any supplemental material intended for posting by ASM should be uploaded with their legends separate from the main manuscript. You can combine all supplemental material into one file (preferred) or split it into a maximum of 10 files with all associated legends included.

For complete guidelines on revision requirements, see our Submission and Review Process webpage. Submission of a paper that does not conform to guidelines may delay acceptance of your manuscript.

Data availability: ASM policy requires that data be available to the public upon online posting of the article, so please verify all links to sequence records, if present, and make sure that each number retrieves the full record of the data. If a new accession number is not linked or a link is broken, provide Spectrum production staff with the correct URL for the record. If the accession numbers for new data are not publicly accessible before the expected online posting of the article, publication may be delayed; please contact production staff (Spectrum@asmusa.org) immediately with the expected release date.

Publication Fees: For information on publication fees and which article types are subject to charges, visit our website. If your manuscript is accepted for publication and any fees apply, you will be contacted separately about payment during the production process; please follow the instructions in that e-mail. Arrangements for payment must be made before your article is published.

ASM Membership: Corresponding authors may join or renew ASM membership to obtain discounts on publication fees. Need to upgrade your membership level? Please contact Customer Service at Service@asmusa.org.

The ASM Journals program strives for constant improvement in our submission and publication process. Please tell us how we can improve your experience by taking this quick Author Survey.

Thank you for submitting your paper to Spectrum.

Sincerely,
Weiqiang Chen
Editor
Microbiology Spectrum

This manuscript evaluates the performance of MxA-based RDT, combined with pathogen sequencing, an approach highly relevant in resource-limited settings with frequent diagnostic uncertainty. The authors also explore other incidental causes in MxA-positive samples. While the study is of interest, a few concerns should be addressed.

1. In this manuscript, the authors are expected to report the level of agreement between MXA-RDT and MXA-ELISA using kappa statistics based on predefined cutoff values.
2. In subsection 4.3 of the Results, the non-specific nature of MxA may lead to an overestimation of positivity in MxA RDT when only DENV and CHIKV PCR are used as references. The authors may want to justify this approach and assess MxA RDT performance using combined PCR and sequencing data as a reference.
3. Typographic errors should be corrected—for example, the initials of the paragraph in section 4.6.2 (lines 423 and 424).
4. The source (Word file) does not have line numbers.

Response to Reviewers

Manuscript: **Kane et al. (Spectrum03392-25)**
Title: Evaluating Myxovirus Resistance Protein A-Based Rapid Testing Combined with Pathogen Sequencing for Arboviral and Incidental Viral Infection Surveillance in Senegal.

We thank the Editor and the Reviewers for their careful assessment and constructive comments. We have revised the manuscript accordingly and provide below a point-by-point response. Reviewer comments are reproduced in italics; our responses follow in regular text. We indicate where changes were made in the revised manuscript (section references and line numbers in the marked-up copy).

Reviewer 1

Comment 1 (Major)

The retrospective design and use of outbreak samples limit the generalizability to routine clinical care. Patient selection criteria and relevant clinical metadata are not fully described, which may introduce bias and confounders affecting MxA levels. Prospective studies including systematically confirmed bacterial and parasitic infections as controls are needed to rigorously assess diagnostic specificity.

Response:

Thank you. We agree. We clarified the retrospective sampling framework and the available clinical metadata (age, sex, and symptom-onset date when reported), while acknowledging the limits of outbreak-response sampling and variable metadata completeness.

To partially address potential parasitic confounding in this malaria-endemic setting, we performed pan-Plasmodium PCR on a subset of archived specimens with sufficient residual volume (n = 68). Nineteen were malaria PCR-positive, and none of these malaria-positive specimens were MxA-positive by RDT or ELISA, nor DENV/CHIKV RT-qPCR–positive, arguing against malaria as a driver of MxA positivity in this dataset.

We also fully agree that prospective studies with systematically confirmed bacterial and parasitic infections as controls are needed to rigorously assess diagnostic specificity. In this retrospective cohort, samples were collected as mild febrile presentations during arboviral surveillance and we only had blood specimens; comprehensive bacterial confirmation would typically require broader specimen types and a dedicated clinical microbiology work-up. Moreover, within a blood-only design, detection of bacteria would more commonly reflect bacteremia and potentially more severe syndromes (e.g., sepsis), which were not the focus of these outbreak investigations. We have strengthened the Discussion accordingly and highlighted the need for well-characterized bacterial and parasitic control panels in future prospective validation.

Changes made in manuscript:

Methods: We expanded the “Study Design and Sample Panels” section to better describe the retrospective selection strategy and the clinical metadata available (age, sex, and symptom-onset date when reported). We also clarified sample handling, anonymization/de-identification procedures, and the rationale for conducting additional malaria testing in a malaria-endemic setting using residual specimens with sufficient remaining volume.

Results/Discussion: We added the pan-Plasmodium PCR subset analysis and tried to strengthen the limitations and future prospective validation statements, emphasizing the need for systematically confirmed bacterial and parasitic control panels to rigorously assess diagnostic specificity.

Comment 2 (Major)

The reported moderate sensitivity and specificity (~70%) of the MxA RDT indicate promise but are below ideal diagnostic thresholds. The virus-specific thresholds are a useful step; however, the manuscript should discuss more clearly the clinical implications of this moderate accuracy and the need for multiplex biomarker panels to improve performance, especially in co-infections or immunocompromised patients.

Response:

Thank you for this important point. We agree that the observed performance (~70% sensitivity/specificity) is promising but below ideal thresholds for a stand-alone diagnostic. We therefore expanded the Discussion to clarify the intended clinical role of the MxA RDT as a frontline triage/prioritization tool—to support early decision-making (e.g., referral and prioritization for confirmatory RT-qPCR and/or sequencing) rather than to provide definitive etiologic attribution on its own.
We also strengthened the discussion of how multiplex host-response panels could improve discrimination across heterogeneous febrile syndromes by combining interferon-driven markers (e.g., MxA) with inflammation-associated markers, and highlighted why such approaches are particularly relevant in co-infections and in patients with altered immune responses (e.g., immunocompromised individuals), where interferon signatures may be blunted or non-specific.

Changes made in manuscript:

Discussion: We added a dedicated paragraph interpreting the clinical implications of moderate accuracy, clarified the intended use-case as triage/prioritization (not a stand-alone etiologic test), and expanded the forward-looking section on multiplex biomarker strategies, including considerations for co-infections and immunologically vulnerable populations.

Comment 3 (Major)

While the use of genomic sequencing to identify missed viral pathogens in MxA-positive but RT-PCR-negative samples is a strong point, the detection of Torque teno virus underscores a limitation: elevated MxA may reflect nonspecific interferon responses unrelated to acute infection. This diagnostic ambiguity requires further investigation through additional molecular profiling and clinical correlation.

Response:

We agree and have tempered the interpretation accordingly. We now explicitly state that MxA is a non–pathogen-specific marker of type I interferon activation, and that the detection of TTV (and Parvovirus B19) in MxA-positive/arbovirus RT-qPCR–negative samples may reflect background anellovirus carriage, incidental viral detection, and/or non-specific interferon activation rather than the primary cause of the febrile episode. We also highlight this as an important source of diagnostic ambiguity and outline next steps to address it, including prospective clinical adjudication, broader pathogen testing, and integrated clinical and host-response profiling.

Changes made in manuscript:

We revised the wording in the "Incidental Viruses Detected" section to add a more cautious interpretation and to emphasize the non-specific nature of MxA. We also expanded the limitations and future directions to propose prospective validation with clinical correlation, broader pathogen panels, and host-response profiling to better distinguish incidental findings from causative infections in MxA-positive/RT-qPCR–negative cases.

Comment 4 (Major)

The discussion would benefit from deeper consideration of practical challenges for implementing MxA diagnostics in low-resource settings, including supply chain logistics, training, quality control, and integration into existing health and surveillance infrastructures, to better inform real-world applicability.

Response:

We agree with the reviewer. We expanded the Discussion to better address on-the-ground deployment issues for MxA diagnostics in low-resource settings. Beyond cost-effectiveness considerations, we added practical points on product availability and distribution, staff training, and feasible quality safeguards. We also clarified how results can be captured and routed through existing fever pathways, referral systems, and routine surveillance reporting, and positioned MxA within a tiered workflow (POC triage → confirmatory molecular testing → sequencing for selected cases).

Changes made in manuscript:

Discussion: We expanded the paragraph beginning with the health economic analyses/cost-effectiveness statement to incorporate concrete implementation considerations for low-resource settings, including supply continuity and logistics (storage and lot traceability), staff training, pragmatic QA/QC measures (incoming lot checks, reader checks where applicable, tracking invalid results, and repeat/escalation rules for borderline or discordant results), as well as result documentation/reporting and integration into existing fever pathways, referral networks, and routine surveillance workflows.

Comment 1 (Minor)

The methods section should clarify sample storage conditions and steps taken to minimize assay variability or batch effects.

Response:

We share the reviewer's view. While the manuscript already stated that archived specimens were stored at −80 °C, we added additional details on pre-analytical handling and steps used to limit variability and potential batch effects. We now specify that, for MxA testing, samples were thawed once under a standardized workflow, gently mixed for homogeneity, and aliquoted to avoid additional freeze–thaw cycles. We also clarify that both the MxA RDT and ELISA were performed according to standardized manufacturer protocols with duplicate measurements for every specimen, and that RDT readout was standardized using the Bi-Reader. For the ELISA, we noted that

concentrations were reported as the mean of duplicates, supporting reproducibility across runs.

Changes made in manuscript:
In the first three subsections of the Materials and Methods , we described the procedures used to limit assay variability, including standardized thawing/mixing/aliquoting to reduce freeze–thaw–related effects, duplicate measurements for both RDT and ELISA, consistent Bi-Reader readout for the RDT, and a standardized ELISA workflow including full standard curves.

Comment 2 (Minor)

Adding a schematic figure illustrating the workflow combining MxA testing with metagenomic sequencing could improve reader understanding.

Response:
We fully agree. We added the requested workflow schematic that lays out the study pipeline from routine DENV/CHIKV RT-qPCR diagnosis (pre-study) to MxA RDT/ELISA testing, followed by selection of discordant cases for sequencing (metagenomic + hybrid-capture) and final interpretation.

Changes made in manuscript:
We added workflow schematic as Figure S1 and cited it in Section 3.4. As a result, prior Supplementary Figures S1–S3 have been renumbered to S2–S4.

Comment 3 (Minor)

Please clarify if MxA testing was performed blinded to RT-PCR and sequencing results to avoid bias.

Response:
Thank you to point this out. We clarified this in the Methods. Because the sample panels were defined upfront based on prior DENV/CHIKV RT-qPCR status, blinding was not possible at the panel-selection stage. However, specimens were relabeled with study IDs and the operators performing the MxA RDT/ELISA were blinded to individual RT-qPCR results during testing. Sequencing was performed only after completion of MxA testing and therefore did not influence MxA readouts. The sequencing subset was subsequently defined based on discordance (MxA RDT-positive with DENV/CHIKV RT-qPCR-negative status).

Changes made in manuscript:
In the Materials and Methods section, we added explicit statements on panel definition and operator blinding in Section 3.1, and clarified sequencing timing and discordance-based subset selection in Section 3.4.

Comment 4 (Minor)

Consistency in terminology between "cut-off" and "threshold" is recommended for clarity; minor typographical and grammatical errors should be addressed.

Response:
Thank you for this helpful suggestion. We harmonized terminology across the manuscript by using “cut-off” for diagnostic decision values and reserving “threshold” for standard expressions such as RT-qPCR Ct (cycle threshold). We also carefully re-read the manuscript and tried to correct minor typographical, grammatical, and formatting issues to improve overall readability.

Changes made in manuscript:
We standardized “cut-off” versus “threshold” usage across the text, tables, and figure legends, and fixed minor typos, grammar, and formatting to improve clarity and consistency. All edits are visible in the tracked-changes version of the revised manuscript.

Comment 5 (Minor)

A brief discussion on potential MxA induction in non-viral inflammatory conditions and its impact on specificity would strengthen the manuscript.

Response:

We agree and have expanded the Discussion to highlight that MxA is a marker of type I interferon activation, which can be induced by infections beyond the targeted arboviruses and, less commonly, by non-infectious inflammatory states. We note that this biology can reduce the apparent specificity when the reference standard is restricted to DENV/CHIKV RT-qPCR, and we link this point to the malaria subset analysis and to the need for prospective cohorts with broader etiologic confirmation. These edits are visible in the tracked version of the manuscript.

Changes made in manuscript:
In the paragraph on the limitations of our study, we added a short clarification on non-specific interferon/MxA induction outside DENV/CHIKV and its implications for specificity estimates when using a limited RT-qPCR reference standard, placed immediately after the statement that controls were not systematically confirmed and before the malaria subset sentence.

Reviewer 2

Comment 1

In this manuscript, the authors are expected to report the level of agreement between MXA-RDT and MXA-ELISA using kappa statistics based on predefined cutoff values.

Response:

Thank you for this very helpful and critical suggestion. We now report Cohen's kappa (unweighted) to quantify qualitative agreement between MXA-RDT and MXA-ELISA using the predefined manufacturer cut-offs (RDT 12 ng/mL; ELISA 10 ng/mL). Using the 169 paired results available, qualitative agreement was 83.4% and $\kappa = 0.67$, indicating moderate-to-substantial agreement beyond chance. While implementing the kappa analysis, we also identified and corrected minor inconsistencies in the agreement/performance calculations, ensuring the reported metrics are now fully consistent across the Results, Figure 1, and the Supplement. We also added a brief statement in the Discussion highlighting the moderate-to-substantial agreement between platforms, supporting the operational relevance of the RDT in settings where ELISA is not feasible.

Changes made in manuscript:

Results and Table S1: added κ and 2×2 table based on predefined cut-offs.
We have included the kappa method using the predefined cut-offs in the Statistical Analysis subsection. Also, in subsection 4.2, we have updated the qualitative agreement statement (83.4%, n = 169 paired samples), revised Figure 1 accordingly, added a short agreement statement in the Discussion, and aligned the related agreement/performance metrics throughout the manuscript (all edits are visible in the tracked version).

Comment 2

In subsection 4.3 of the Results, the non-specific nature of MxA may lead to an overestimation of positivity in MxA RDT when only DENV and CHIKV PCR are used as references. The authors may want to justify this approach and assess MxA RDT performance using combined PCR and sequencing data as a reference.

Response:

We acknowledge this issue and have addressed it. We clarified that DENV/CHIKV RT-qPCR was used as the primary reference standard because this evaluation was embedded within two confirmed outbreak investigations with prespecified diagnostic targets. At the same time, we acknowledge that MxA is a non–pathogen-specific type I interferon marker, so relying only on DENV/CHIKV RT-qPCR can classify MxA-positive samples driven by other etiologies as "false positives," lowering the apparent specificity. To address this concern, we added a sequencing-informed adjudication of selected discordant cases and performed a complementary sensitivity analysis using an expanded reference that incorporates sequencing results for MxA-positive/RT-qPCR-negative samples. This provides a more nuanced interpretation of discordant results and helps contextualize performance estimates beyond the targeted RT-qPCR panel.

Changes made in manuscript:
We added text introducing the expanded-reference sensitivity analysis incorporating sequencing adjudication of selected discordant samples, and describing the sequencing workflow and findings used for this adjudication (all edits visible in the tracked version). We have also included a schematic workflow as a new supplementary Figure S1 to make sure that the study design is clear enough.

Comment 3

Typographic errors should be corrected—for example, the initials of the paragraph in section 4.6.2 (lines 423 and 424).

Response:
We agree and have corrected the typographic errors noted by the reviewer, including the duplicated/erroneous wording at the beginning of the paragraph in section 4.6.2 (lines 423–424). In addition, we harmonized virus nomenclature throughout the manuscript so that Parvovirus B19 is introduced once and subsequently referred to consistently as B19V, with abbreviations standardized after first use. All corrections are reflected in the tracked version.

Changes made in manuscript:
Mainly in the subsection 4.6.2 and the Discussio section, we corrected the typographical error at the start of the paragraph (lines 423–424) and standardized virus naming/abbreviations (Parvovirus B19 → B19V after first mention), along with minor formatting consistency edits visible in the tracked manuscript.

Comment 4

The source (Word file) does not have line numbers.

Response:
As requested, in addition to this point-by-point Response to Reviewers, we have prepared a revision package that includes a marked-up version of the manuscript with continuous line numbering, as well as a clean revised version, in line with the journal's requirements.

Re: Spectrum03392-25R1 (**Evaluating Myxovirus Resistance Protein A-Based Rapid Testing Combined with Pathogen Sequencing for Arboviral and Incidental Viral Infection Surveillance in Senegal.**)

Dear Dr. Moussa Moise DIAGNE:

Your manuscript has been accepted, and I am forwarding it to the ASM production staff for publication. Your paper will first be checked to make sure all elements meet the technical requirements. ASM staff will contact you if anything needs to be revised before copyediting and production can begin. Otherwise, you will be notified when your proofs are ready to be viewed.

Data Availability: ASM policy requires that data be available to the public upon online posting of the article, so please verify all links to sequence records, if present, and make sure that each number retrieves the full record of the data. If a new accession number is not linked or a link is broken, provide production staff with the correct URL for the record. If the accession numbers for new data are not publicly accessible before the expected online posting of the article, publication may be delayed; please contact ASM production staff immediately with the expected release date.

Publication Fees: For information on publication fees and which article types have charges, please visit our website. We have partnered with Copyright Clearance Center (CCC) to collect author charges. If fees apply to your paper, you will receive a message from no-reply@copyright.com with further instructions. For questions related to paying charges through RightsLink, please contact CCC at ASM_Support@copyright.com or toll free at +1-877-622-5543. CCC makes every attempt to respond to all emails within 24 hours.

ASM Membership: Corresponding authors may join or renew ASM membership to obtain discounts on publication fees. Need to upgrade your membership level? Please contact Customer Service at Service@asmusa.org.

Embargo Policy: A press release may be issued as soon as the manuscript is posted on the Spectrum Latest Articles webpage. The corresponding author will receive an email with the subject line "ASM Journals Author Services Notification" when the article is available online.

The ASM Journals program strives for constant improvement in our submission and publication process. Please tell us how we can improve your experience by taking this quick Author Survey.

Thank you for submitting your paper to Spectrum.

Sincerely,
Weiqiang Chen
Editor
Microbiology Spectrum

Reviewer #2 (Comments for the Author):

The methods used, results, and conclusions are rigorous.
